# Optimizing Tree Selection for Sustainable Logging in Amazonian Areas with Species Restrictions Using Bio-Inspired Algorithms

**DOI:** 10.3390/biomimetics11100698

**Published:** 2026-10-01

**Authors:** Sebastian Grajeda, Manuel Tupia, Rony Cueva

**Affiliations:** Department of Engineering, Pontificia Universidad Católica del Perú, Av. Universitaria 1801, San Miguel, Lima 15086, Peru; s.grajeda@pucp.edu.pe (S.G.); cueva.r@pucp.edu.pe (R.C.)

**Keywords:** Artificial Bee Colony, genetic algorithm, combinatorial optimization, sustainable forest management, Amazonian tree selection

## Abstract

Forest management planning in the Peruvian Amazon must reconcile economic return, ecological sustainability, spatial coherence, and strict regulatory compliance within a single harvest decision. Biomimetic optimization—search that transposes natural mechanisms such as honeybee foraging, biological evolution, and physical annealing into computation—offers a promising route to such constrained combinatorial problems. This study proposes a set-theoretic discrete adaptation of the Artificial Bee Colony algorithm (Set-ABC), a swarm-intelligence metaheuristic inspired by the division of labor among employed, onlooker, and scout bees, for parcel-based tree selection; food sources are represented as sets of parcel identifiers and neighborhood search is driven by set-algebraic operators rather than continuous or binary encodings. The task is formalized as a regulated NP-hard combinatorial optimization with hard constraints anchored in Law No. 29763 and Supreme Decree No. 018-2015-MINAGRI, plus a penalized species-retention constraint, under an objective maximizing net economic return in monetary units (PEN). Set-ABC is benchmarked against three further nature-inspired paradigms—a Binary ABC (BABC), a Binary Genetic Algorithm (GA), and Simulated Annealing (SA)—on a semi-synthetic instance of approximately 90 parcels and 12 commercial species from a real operational inventory. After Grid Search calibration, the methods were evaluated over 20 paired runs. A Friedman test with Bonferroni-corrected Wilcoxon post hoc comparisons revealed significant differences across all pairs: GA attained the highest mean net return (1,059,364 PEN) and Set-ABC the lowest (983,873 PEN) but the second-most stable variance, establishing this biomimetic set-based formulation as a viable, stable contender for regulated parcel-based forest optimization.

## 1. Introduction

The Amazon basin concentrates one of the largest reserves of commercial timber and standing carbon on the planet, and its management under sustainable-yield regimes is simultaneously an economic activity and a conservation obligation [1,2]. In the Peruvian Amazon, harvest planning is not a purely economic exercise: it is bounded by a national regulatory framework that a valid management plan must satisfy. The Forest and Wildlife Law (Law No. 29763) mandates minimum primary-forest retention, canopy-cover thresholds, and seed-tree preservation ratios, while the national CITES registry restricts the extraction of protected flora species [3,4,5]. A harvest proposal that maximizes revenue but breaches these thresholds is not merely suboptimal—it is legally inadmissible. Planning must therefore reconcile economic return, ecological sustainability, spatial coherence of the harvest block, and strict regulatory compliance within a single decision.

Despite this complexity, a substantial share of harvest planning in the region is still carried out through manual procedures or empirical criteria, which limits the exploration of alternative management scenarios and introduces calculation errors and delays [6,7]. The underlying difficulty is combinatorial: when the concession is discretized into spatial decision units—here, forest parcels aggregating multiple inventoried trees—the task of selecting which parcels to harvest becomes an NP-hard combinatorial optimization problem, structurally related to the Set Covering and Multidimensional Knapsack problems [8,9]. The number of candidate subsets grows exponentially with the number of parcels, and the feasible region is further fragmented by non-linear spatial requirements such as connectivity of the harvest block and tolerance limits on internal enclaves [9,10]. Because the input data (timber volumes, canopy estimates, species inventories) are themselves derived from sampling and carry uncertainty, exact methods stagnate computationally on realistic instances, motivating the use of approximate, metaheuristic search [7,11].

The metaheuristic literature applied to spatial forest planning has followed two main lines. Single-solution strategies—GRASP, Tabu Search, and Simulated Annealing—have proven efficient for coverage and infrastructure-allocation problems but are highly sensitive to neighborhood design and prone to local optima under overlapping constraints [9,10,11]. Population-based and bio-inspired methods, and in particular Genetic Algorithms (GA), have shown stronger global exploration for multi-objective tree selection and seed-tree preservation [12,13,14]. However, a specific gap persists: collective swarm-intelligence algorithms, and the Artificial Bee Colony (ABC) algorithm in particular, have not been adapted to the discrete, parcel-based tree-selection problem, nor systematically benchmarked against established population-based and single-solution metaheuristics on this class of regulated, spatially constrained forest problems. ABC is well established in continuous domains and in some binary reformulations [15,16,17,18], yet its cooperative balance between scout-bee exploration and employed/onlooker-bee exploitation remains untested in this setting.

Methodologically, this work is grounded in biomimetics: the transfer of problem-solving strategies observed in nature into computational search. The three metaheuristic families examined here are all biomimetic in origin—swarm intelligence, in which the Artificial Bee Colony (ABC) algorithm emulates the cooperative foraging of a honeybee colony through the division of labor among employed, onlooker, and scout bees; evolutionary computation, in which the Genetic Algorithm (GA) reproduces the dynamics of natural selection, crossover, and mutation; and physical-process analogs, in which Simulated Annealing (SA) mimics the controlled cooling of a solid to escape local optima. This biomimetic paradigm is a still-active line of research: recent studies continue to refine bee-inspired search through improved ABC variants with new crossover, lock, and feature-selection mechanisms [15,16], alongside other nature-inspired optimizers such as the Artificial Electric Field Algorithm [19].

This biomimetic machinery has proven particularly well suited to sustainable forest management, where the search space is large, nonlinear, and heavily constrained. Multi-objective genetic algorithms have been applied to seed-tree selection and to balancing timber yield against ecosystem services [12,13], and related optimization techniques have supported thinning and harvest decisions from multi-platform inventory data [8]. Within this biomimetic toolkit, however, the honeybee-foraging paradigm remains unexplored for discrete, parcel-based tree selection. The present study addresses that gap by contributing a biomimetic adaptation—Set-ABC—that reformulates the bees’ foraging and perturbation behavior through set-algebraic operators, and by situating it against three other biomimetic paradigms (BABC, GA, and SA) under a unified, regulation-aware evaluation.

Addressing this gap, the present work develops a set-theoretic discrete adaptation of the ABC algorithm (Set-ABC) in which food sources are represented as sets of parcel identifiers and neighborhood construction is governed by set-theoretic operators rather than continuous vector arithmetic or binary bit-masks [20,21]. Set-ABC and three reference metaheuristics—a Binary ABC (BABC), a binary GA, and Simulated Annealing (SA)—optimize a common objective expressed directly in net economic return (PEN), combining gross timber revenue and extraction cost while discounting statutory species-retention infractions through a penalty term, and assigning zero fitness to any solution that violates a hard constraint. The four methods are calibrated under an identical single-round Grid Search protocol and compared over 20 independent runs with matched random seeds, using a paired statistical pipeline (Friedman test with Bonferroni-corrected Wilcoxon post hoc comparisons). The main contributions are therefore: (i) a formal model of regulated parcel-based Amazonian tree selection with hard and penalized constraints anchored in Peruvian legislation; (ii) an operationally interpretable objective function expressed in monetary units that couples economic return with legal compliance; (iii) a novel set-theoretic discrete ABC adaptation driven by set-algebraic operators; and (iv) a statistically rigorous four-way benchmark of the proposed Set-ABC against BABC, GA, and SA on a semi-synthetic instance derived from a real operational concession, which identifies a well-tuned GA as the reference method to beat while establishing Set-ABC as a stable and feasible contender.

This paper is organized as follows. Section 2 reviews related work on optimization for spatial forest planning. Section 3 formalizes the problem, defining decision variables, hard and penalized constraints, and the objective function. Section 4 details the design of the four metaheuristics: the set-theoretic discrete ABC, the binary ABC, the binary GA, and simulated annealing. Section 5 describes the software architecture, the semi-synthetic dataset, the hyperparameter calibration protocol, and the statistical comparison pipeline. Section 6 reports empirical results, and Section 7 discusses their interpretation and limitations. Section 8 presents conclusions and future research directions.

## 2. Related Work

The application of optimization techniques in forest management has progressively expanded from primarily economic and timber-production objectives toward comprehensive frameworks that integrate ecological sustainability, regulatory requirements, and biodiversity conservation [1,6,8]. Forest harvest planning continues to face significant operational challenges because many planning activities are still carried out through manual procedures or empirical decision-making, limiting the evaluation of alternative management scenarios [6]. This traditional practice leads to calculation errors, significant delays, and an inability to process the massive combinatorial explosion of variables within reasonable execution times [7]. Spatial optimization problems in forestry are commonly formulated by discretizing the landscape into spatial decision units, such as management stands, production units, or grid cells, to represent large forest areas and enable the application of combinatorial optimization techniques [9,10,12]. Following this modeling paradigm, the present work adopts forest parcels as the spatial decision units, where each parcel aggregates multiple inventoried trees into a single optimization entity [8,12]. Under this representation, tree selection for harvesting or seed-tree preservation becomes a highly complex NP-hard combinatorial optimization problem, closely related to the Set Covering Problem and the Multidimensional Knapsack Problem [8,9].

In specialized literature, spatial forest planning has incorporated various ecological constraints to prevent inefficient decisions that cause ecosystem fragmentation or vulnerability during environmental audits [2,22]. Multiple authors emphasize that spatial cohesion and forest connectivity require harvest blocks to form connected subgraphs, avoiding isolated intervention zones [9,10]. Simultaneously, models must ensure project economic viability by exceeding fixed operational costs while strictly guaranteeing legal thresholds [6,23].

Although these constraints are commonly addressed in the literature from a general forest management perspective, their application in the Peruvian Amazon requires compliance with the national regulatory framework. Harvest planning must satisfy the provisions of the Peruvian Forest and Wildlife Law (Law No. 29763) and the national CITES registry for protected flora species, while also meeting sustainability requirements such as maintaining minimum primary forest retention, canopy cover, and seed-tree preservation ratios [3,4,5]. Additionally, models must manage enclave tolerance by limiting the number of unharvested parcels inside an active block to avoid disrupting access and transport logistics [6].

Because exact methods suffer from computational stagnation when scaled to real-world instances, research has increasingly turned to metaheuristic algorithms [7,11,19]. Single-solution strategies such as Greedy Randomized Adaptive Search Procedure (GRASP), Tabu Search (TS), and Simulated Annealing (SA) have demonstrated efficiency in discovering near-optimal solutions for forest coverage and infrastructure allocation [9,10]. However, these single-solution metaheuristics show high sensitivity to neighborhood structure design and frequently get trapped in local optima when handling overlapping constraints [7]. Conversely, population-based and bio-inspired metaheuristics, primarily Genetic Algorithms (GA), have shown greater global exploration capacity to optimize harvesting while maintaining landscape resilience and ecosystem services [12,13,14].

Despite these advances, a fundamental technical gap remains in spatial forest selection [7,8]. While GA implementation continues to be widely applied in multi-objective tree selection and seed-tree preservation [12,13], its reliance on rigid binary chromosome representations frequently generates infeasible solutions when handling non-linear spatial constraints, requiring heavy penalty functions that degrade search efficiency [12,13]. Furthermore, there is a distinct absence of studies applying collective swarm intelligence algorithms, specifically the Artificial Bee Colony (ABC) algorithm, to discrete parcel-based tree selection problems. Swarm-based bio-inspired techniques are widely recognized for their natural mechanism balancing global exploration by scout bees and local exploitation by employed and onlooker bees through cooperative foraging behavior [15,16]. Therefore, developing a discrete adaptation of the ABC algorithm based on set-theoretic operators rather than binary vectors constitutes a novel approach for solving parcel-based tree selection problems under the regulatory and ecological constraints that characterize sustainable forest management in the Peruvian Amazon [3,4,6,15].

## 3. Problem Formulation

This section formalizes the spatial tree selection problem for sustainable Amazonian harvesting. The objective is to identify an optimal subset of land parcels that maximizes economic yield and harvested area while satisfying non-linear ecological, regulatory, and spatial constraints.

### 3.1. Decision Variables, Parameters, and Sets

The underlying spatial structure of a forest concession is defined as a graph G = (V, E), where V= {1, 2, 3, ..., N} represents the set of total land parcels and E denotes the list of contact relationships between parcels. A candidate solution X ⊆ V corresponds to the subset of selected parcels chosen for harvesting, containing |X| selected parcels. The fundamental parameters and sets governing the model are defined as follows:

- Parcel Attributes: For each parcel$\;i\;\in \;V,\;a_{i}$ denotes its land area, $c_{i}$ represents the canopy cover area, $c_{i}^{nat}$ represents the canopy cover area of primary/native trees, and $p_{i}\;\in \;\{0,\;1\}$ indicates whether the parcel is designated as a protected area.
- Species Attributes: S= {1, 2, 3, ...,|S|}  represents the set of inventoried tree species. For each species $s\;\in \;S,\;v_{s}$ is the commercial timber value per volume unit, $c_{s}$ is the extraction cost per volume unit, $p_{s}$ is the penalty per unit of volume exceeded, $Q_{s}$ is the total initial tree count across the concession, $W_{s}$ is the total concession wood volume of species s, and $r_{s}\in \;[0,\;1]$ is the mandatory conservation ratio.
- Parcel-Species Relations: $q_{i,s}$ denotes the number of trees and $w_{i,s}$ denotes the timber volume of species s within parcel i.
- Concession Boundaries: $S_{total}$ represents the total concession land area, $C_{total}$ is the initial total canopy area, and $C_{total}^{nat}$ is the initial total primary canopy area.
- Global Parameters: $B_{proj}$ is the project budget that must cover extraction costs, $R_{CC}$ is the minimum required canopy coverage ratio [24], $R_{FC}$ is the minimum required primary forest conservation ratio, $N_{U}$ is the maximum allowable unharvested internal parcels [24], $G_{max}$ is the theoretical maximum revenue if all parcels were harvested, and $A_{max}\;=\;S_{total}\;$is the maximum harvestable area.

### 3.2. Constraints and Solution Feasibility

A solution X ⊆ V is subject to two classes of constraints: hard constraints (which render a solution completely infeasible with F(X) = 0) and penalized constraints (which progressively degrade solution fitness).

#### 3.2.1. Hard Constraints (Feasibility Boundary)

If any of the following six conditions are violated, the fitness evaluation immediately assigns F(X) = 0:

1. Spatial Connectivity: The sub-graph induced by the selected parcels X must form a single connected component. Evaluated via Breadth-First Search (BFS) over the list of contact relationships between parcels E, X must satisfy |X| ≥ 3 and BFS(X) = |X|.
2. Internal Enclave Limit: Complete spatial compactness around non-harvestable or protected units is often practically unachievable. Therefore, an operational tolerance threshold $N_{u}$ is defined for the number of unharvested parcels embedded within the perimeter polygon generated by X, denoted as H(X). This mechanism provides the spatial flexibility required for the metaheuristics to naturally bypass or contour around protected zones without triggering immediate disqualification, while preventing the formation of excessively hollow, fragmented, or structurally inefficient harvest blocks:

(1) $${H(X)\;\leq N}_{u}$$

3. Exclusion Zones Protection: National forestry regulations strictly forbid timber extraction within ecologically sensitive areas, riparian buffers, steep slopes, or officially declared conservation cores pursuant to Supreme Decree No. 018-2015-MINAGRI [24] and Law No. 29763 [3]. Consequently, no parcel designated as an exclusion/protected unit ($p_{i}\;=\;1$) can be incorporated into X.

(2) $$\sum_{i\;\in \;X}p_{i}\;=0$$

4. Economic Viability: A forest intervention must remain financially viable within allocated capital boundaries; thus, the total wood extraction C(X) incurred by harvesting the selected parcels must not exceed the available project budget $B_{proj}$.

(3) $$C(X)=\;\sum_{i\;\in \;X}\sum_{s\;\in \;S}\left(c_{s}\cdot w_{i,s}\right)<B_{proj}$$

5. Minimum Forest Canopy Coverage Constraint: Pursuant to the statutory definition of forest ecosystems established in Supreme Decree No. 018-2015-MINAGRI, post-harvest residual tree cover must retain at least the fraction *R*_*CC*_ = 0.25 of the initial concession canopy area *C*_*total*_. This guarantees that canopy opening does not exceed the legal threshold that distinguishes managed productive forest from land-use degradation [24]:

(4) $$\frac{C_{total}\;-\sum_{i\;\in \;X}c_{i}}{C_{total}\;}\;\geq \;R_{CC}$$

6. Primary Forest Conservation Constraint: Forest management in native Amazonian stands is bounded by statutory sustainability criteria governed by Law No. 29763 and the regulations approved under Supreme Decree No. 018-2015-MINAGRI [24], which mandate the preservation of natural forest structure, seed sources, and standing ecological biomass. To avoid permanent ecosystem depletion and severe administrative infractions evaluated under OSINFOR oversight guidelines [25], harvest operations must retain at least the legal fraction *R*_*FC*_ = 0.30 of the original native/primary forest canopy cover *C*_*totalnat*_. Feasible solutions must strictly satisfy:

(5) $$\frac{C_{total}^{nat}\;-\sum_{i\;\in \;X}c_{i}^{nat}}{C_{total}^{nat}}\;\geq \;R_{FC}$$

#### 3.2.2. Penalty Constraints

Unlike hard constraints, penalty constraints enforce ecological conservation thresholds rooted in Peruvian forestry legislation (Law No. 29763) and regulatory guidelines established by OSINFOR [3,25]. Specifically, OSINFOR’s oversight methodology penalizes infractions based on species threat status and ecological value. To reflect these standards, a Species Retention Constraint is considered: for each species s ∈ S, the proportion of remaining unharvested trees relative to their initial population $Q_{s}$ must meet or exceed the species conservation ratio $r_{s}$:

(6) $$\frac{Q_{s}\;-\;\sum_{i\;\in \;X}q_{i,s}}{Q_{s}}\;\geq r_{s},\;\forall \;s\;\in \;S$$

Violating this threshold does not invalidate the candidate solution; rather, an indicator function identifies the breached boundary to compute the extent of non-compliance. The formal mathematical formulation of this penalty function, along with its weighting and integration into the overall objective function, is detailed in Section 3.3.

### 3.3. Objective Function and Design Justification

The objective function F(X)∈ [0, +∞[ evaluates solution quality based on economic profitability while strictly enforcing regulatory, spatial, and budgetary viability. The proposed objective function directly models the net economic return (in monetary currency, PEN) adjusted by linear economic penalties for species-retention infractions.

Formally, for a candidate harvest block X ⊆ V, the objective value is computed as:

(7) $$F(X)\;=\;max(0,\frac{\left\lfloor 100\cdot (G(X)\;-C(X)\;-\;P(X))\right\rfloor }{100})$$

where

○ G(X) represents the total gross timber revenue obtained from the harvested parcels:

(8) $$G(X)=\;\sum_{i\;\in \;X}\sum_{s\;\in \;S}\left(v_{s}\cdot w_{i,s}\right)$$

○ *C*(*X*) is the total operational extraction cost across all harvested units:

(9) $$C(X)=\;\sum_{i\;\in \;X}\sum_{s\;\in \;S}\left(c_{s}\cdot w_{i,s}\right)$$

○ P(X) is the total monetary penalty incurred when the harvested volume of restricted timber species breaches statutory retention quotas (detailed in Section 3.4).
○ The scaling factor $\left\lfloor 100\cdot (\cdot )\right\rfloor /100$ applies a two-decimal floor truncation reflecting financial cent-level precision, and the non-negativity truncation max(0,·) prevents unviable negative returns.

The architectural design of F(X) is justified through two principles:

1. Direct Operational Interpretability: Formulating F(X) in net monetary units provides concession managers and forestry auditors with an immediate, tangible assessment of project profit margins, removing the ambiguity associated with synthetic dimensionless indices.
2. Strict Compliance Guarantee: Solutions breaching hard constraints (such as connectivity, enclave tolerance, exclusion zones, budgetary caps, canopy cover, or primary forest conservation) receive F(X) = 0, guaranteeing that only legally and operationally permissible harvesting schedules can achieve positive fitness.

### 3.4. Formulation of Penalties

Among the ecological restrictions, the species conservation threshold operates with a soft penalty mechanism that penalizes over-harvesting on an economic scale. Let $q_{s}\left(X\right)$ (Equation (10)) be the harvested tree count of species s, and $L_{s}$ (Equation (11)) be the maximum allowable harvested limit.

(10) $$q_{s}(X)=\sum_{i\;\in \;X}q_{i,s}$$

(11) $$L_{s}=\left\lceil Q_{s}\cdot (1-r_{s})\right\rceil$$

If the tree count exceeds the permissible ceiling $q_{s}(X)>L_{s}$, the surplus harvested timber volume $∆W_{s}(X)$ beyond the legal volumetric quota $W_{s}\cdot (1\;-\;r_{s})$ is penalized:

(12) $$∆W_{s}(X)=max(0,\sum_{i\;\in \;X}w_{i,s}\;-\;W_{s}\cdot (1\;-\;r_{s}))$$

The aggregate species violation penalty P(X) is calculated as:

(13) $$P(X)=\sum_{s\;\in \;S\;|\;q_{s}(X)>L_{s}}\left(∆W_{s}(X)\cdot \;p_{s}\right)$$

where $p_{s}$ represents the unit penalty cost assessed per volume unit of timber extracted beyond the sustainable threshold for species s. This penalization rate is grounded in the administrative enforcement framework and official fining methodology established in Presidential Resolution No. 021-2018-OSINFOR [25]. Under these supervisory guidelines, unauthorized or surplus commercial timber extraction is sanctioned through proportional economic fines scaled by the commercial value of the affected species and the volume unauthorizedly harvested. Integrating $p_{s}$ directly into the objective function reflects the regulatory liability of exceeding species-level retention quotas, ensuring that the optimization algorithms heavily discount solutions that breach authorized silvicultural thresholds. If $q_{s}(X)\leq L_{s}$, no penalty is incurred for that species.

## 4. Algorithm Design

This section details the algorithmic framework designed for parcel-based forest optimization under Amazonian regulatory and spatial constraints. To thoroughly evaluate performance across distinct search paradigms and encoding schemes, four metaheuristic strategies are implemented: a novel Set-Theoretic Discrete Artificial Bee Colony (Set-ABC), a Binary Artificial Bee Colony (BABC), a traditional Binary Genetic Algorithm (GA), and a single-solution Simulated Annealing (SA) metaheuristic.

Because uniform random parcel selection yields disjoint subgraphs and violates exclusion restrictions with near certainty, all four approaches are initialized using a specialized graph-based constructive heuristic that guarantees spatial connectivity, exclusion zone compliance, and enclave feasibility. Subsequently, the algorithms explore the combinatorial landscape using either discrete set-algebraic operators (Set-ABC) or bitwise transformations over binary strings (BABC, GA, and SA) to evaluate the relative merits of set-based versus binary solution structures.

### 4.1. Feasible Solution Initialization

Generating feasible initial solutions uniformly at random over a spatial lattice is statistically improbable due to strict topological and ecological constraints: spatial connectivity and exclusion of protected areas. Selecting parcels independently would almost certainly produce disconnected subgraphs or violate protected barriers, leading to degenerate initial states where F(X) = 0.

To supply all evaluated metaheuristics with viable initial points, an iterative, graph-based constructive heuristic is implemented (Algorithm 1):
**Algorithm 1:** Pseudocode for the proposed random generation of solutionInput: n (length proposed of solution), G (All connected parcels) Output: X1:Visited ← InitializeVisitedMap(G,False)//Boolean vector with parcel index2:parcel ← Null3:**while** parcel = Null
**or** is Protected(parcel) **then**4:    parcel ← RandomParcelChoose(G)5:    Visited[parcel] ← True6:**end while**7:Visited[parcel] ← True8:Add(X,parcel)9:**for** counter ← 0 **to** n **then**10:      *N*← GetNeighborhoodOf(X, G)//all neighboring parcels of X without repetition11:      **for each** neighbor **in** N12:          **if not** is Protected(neighbor) **then**13:              Add(X,neighbor)14:                **break**15:          Visited[neighbor] ← True16:**end for**

### 4.2. Set-Theoretic Discrete Artificial Bee Colony (Set-ABC) Adaptation

Standard ABC implementations operate in continuous domains using floating-point vector arithmetic. To address the discrete combinatorial nature of parcel selection without relying on continuous rounding heuristics, this study develops a set-theoretic adaptation of ABC.

#### 4.2.1. Food Source and Nectar Representation

- Nectar Unit: Represents the minimal atomic component of a solution, defined directly as a unique integer parcel identifier rather than a binary bit [17]. This formulation is hypothesized to enhance search performance and solution quality, as manipulating spatial subsets through direct set operators may guide neighborhood exploration more naturally than independent bit-flipping across fixed-length binary strings.
- Food Source ($X_{i}$): Represents a candidate harvest solution composed of a variable-length list of unique parcel identifiers (Equation (14)), its evaluated fitness value $F(X_{i})$, and a visit counter ${trial}_{i}$.

(14) $$X_{i}=\{p_{1},p_{2},\;...\;,p_{k}\}$$

- Colony Population and Search Boundaries: The colony manages a population of SN food sources whose cardinality (number of selected parcels) is bounded by lower and upper operational search boundaries, $L_{inf}$ and $L_{sup}$, respectively:

(15) $$\left|L_{inf}\right|\leq \left|X_{i}\right|\leq \left|L_{sup}\right|$$

- Boundary Violation Replacement and Food Source Exhaustion: During the employed and onlooker bee phases, candidate neighbors $V_{i}$ are generated through set perturbation. If the resulting parcel count of an accepted candidate breaches either operational threshold (Equation (15)), the solution is reset to the corresponding boundary benchmark to enforce spatial feasibility within the designated search window. In parallel, if a food source fails to improve its fitness over a predefined number of consecutive iterations ${trial}_{i}\;\geq \;Limit$, it is abandoned during the scout phase and replaced by a newly constructed feasible solution generated within $\left[\left|L_{inf}\right|,\left|L_{inf}\right|\right]$ via Algorithm 1, resetting ${trial}_{i}$ to 0.

#### 4.2.2. Set-Theoretic Operators and Neighborhood Search

Neighboring food source construction $V_{i}$ from an active food source $X_{i}$ and a randomly chosen food source $X_{k}$ is governed by discrete set algebra rather than continuous vector addition [20]. The discrete operators are defined as follows:

1. Set Addition (⊕): Given parcel sets A and B, C = A⊕B generates the set union A∪B without duplicate parcel identifiers.
2. Set Subtraction (⊖): Given parcel sets A and B, C = A⊖B generates the set difference A∕B, removing all parcels present in B from A.

#### 4.2.3. New Food Source Construction Mechanism

In the standard continuous formulation of the Artificial Bee Colony (ABC) algorithm, a candidate food source $V_{i}$ is generated by perturbing the current solution $X_{i}$ with respect to a randomly selected food source $X_{k}$ using continuous vector arithmetic (Equation (16)), where $\phi_{i,j}\in \;[-1,1]$ is a uniform random number [18]:

(16) $$V_{i,j}\;=\;X_{i,j}+\;\phi_{i,j}\cdot (X_{i,j}\;-X_{k,j})$$

To adapt this mechanism to the discrete parcel-selection domain without relying on continuous rounding heuristics, this study reformulates the perturbation process using set-theoretic operations, similar to the process used in [21]. The logic for generating a new food source is defined as follows (Algorithm 2):
**Algorithm 2:** Pseudocode for the proposed food source constructionInput: i (food source index), SN Output: $V_{i}$(new food source)1:k ← RandomIndex(0,|SN|)2:ϕ ← RandomRealNumber(−1,1)3:$X_{i}$ ← SN[i]4:$X_{k}$ ← SN[k]5:**if** i ≠ k **and** $\left|\phi \right|\;>\;0.01$ **then**6:     only_current ← $X_{i}⊖X_{k}$7:     only_random ← $X_{k}⊖X_{i}$8:     **if** ϕ > 0**then**9:          n ← $\left\lfloor \phi \cdot |only\_random|\right\rfloor$10:          $V_{i}\;$← $X_{i}\;⊕$ RandomNectarChoice(only_random,n)11:     
**else**
12:          n ← $\left\lfloor -\;\phi \cdot |only\_current|\right\rfloor$13:          $V_{i}\;$← $X_{i}\;⊖$ RandomNectarChoice(only_current,n)14:     
**end if**
15:**else**16:          $V_{i}$← $X_{i}$17:**end if**

An important point to highlight is that in lines 5 and 16, having equal indexes or a very low ϕ, we double the original food source to force the ${trial}_{i}$ in the proposed food source evaluation (Algorithm 3) and increase the probability of replacement in the Scout phase (Algorithm 4).

#### 4.2.4. Source Replacement and Bounded Evaluation

Source replacement follows a selection mechanism bounded by parcel search limits. If the newly generated candidate source $V_{i}$ achieves a strictly higher fitness amplitude than $X_{i}$, it replaces the current solution. However, to maintain search feasibility within the designated spatial bounds, if the parcel count of $V_{i}$ breaches either the upper limit or lower limit, the solution is reset to the boundary benchmark. If $V_{i}$ fails to improve fitness,$\;X_{i}$ is retained and its visit counter increments (${trial}_{i}$) [16]. The replacement evaluation logic is defined as follows (Algorithm 3):
**Algorithm 3:** Pseudocode for the proposed food source evaluationInput: $X_{i}$ (current food source), $V_{i}$(new food source), $L_{inf}$(lower limit), $L_{sup}$(upper limit) Output: $V_{i}$(new food source)1:fitness_current ← ${F(X}_{i})$(Equation (7))2:fitness_new ← ${F(V}_{i})$(Equation (7))3:**if** fitness_new > fitness_current **then**4:     **if** $\left|V_{i}|\;>\;|L_{sup}\right|$ **then**5:          $V_{i}$ ← $L_{sup}$6:     
**else**
7:          **if** $\left|V_{i}|\;<\;|L_{inf}\right|$ **then**8:               $V_{i}$ ← $L_{inf}$ **end if**9:     
**end if**
10:**else**11:     ${trial}_{i}$ ← ${trial}_{i}$+ 112:     $V_{i}$ ← $X_{i}$13:**end if**

#### 4.2.5. Overall Set-Theoretic Discrete Artificial Bee Colony Algorithm

The overall execution flow of the adapted Set-Theoretic Discrete Artificial Bee Colony algorithm integrates the food source initialization, the three foraging phases (Employed, Onlooker, and Scout bees), and the global best solution memorization across iterations (Algorithm 4).

- Initialization Phase: Generates an initial colony of Size candidate solutions, where each food source is represented as a random set of unique parcel identifiers bounded by $L_{inf}$(lower limit) and $L_{sup}$(upper limit).
- Employed Bee Phase: Each employed bee explores the discrete neighborhood of its assigned food source by applying set-theoretic operations (⊕ and ⊖) with a randomly selected peer, greedily retaining the candidate solution if its net economic return improves.
- Onlooker Bee Phase: Onlooker bees probabilistically choose promising food sources through roulette wheel selection based on relative fitness and conduct localized set perturbations to exploit high-yield foraging regions.
- Scout Bee Phase: When a food source fails to improve after a predetermined threshold (Limit), it is considered exhausted and abandoned, prompting a scout bee to generate a new random parcel set to prevent search stagnation.
- Memorization/Update Phase: Evaluates the entire updated population at the end of each iteration to record and preserve the global best harvest block ($X_{best},\;f_{best}$) discovered so far.

**Algorithm 4:** Pseudocode for Set-Theoretic Discrete Artificial Bee Colony (Set-ABC)Input: MaxIter, Limit, Size, $L_{inf}$(lower limit), $L_{sup}$(upper limit) Output: $X_{best}$ (best food source),$\;f_{best}$ (best fitness)1:SN ← InitializePopulation($Size,L_{inf},\;L_{sup}$)2:**for** it = 1 to MaxIter **do**3:     **for** i = 1 to |SN| **do//**Employed phase4:          $X_{i}$ ← SN[i]5:          $V_{i}$ ← NewSourceConstruction(i,SN) (Algorithm 2)6:          SN[i] ← FoodSourceEvaluation($X_{i},V_{i},L_{inf},L_{sup}$) (Algorithm 3)7:     **end for**8:     **for** i = 1 to |SN| **do//**Onlooker phase9:          k ← SelectionFoodSourceIndex(“Roulette Wheel”,SN)10:          $V_{k}$ ← NewSourceConstruction(k,SN) (Algorithm 2)11:          SN[k] ← FoodSourceEvaluation($X_{k},V_{k},L_{inf},L_{sup}$) (Algorithm 3)12:     **end for**13:     **For each** $X_{i}$ **with** ${trial}_{i}\geq Limit\;$**do//**Scout phase14:          **do**15:                 n ← RandomPositiveNumber($\left|L_{inf}\right|,\left|L_{sup}\right|$)16:            $X_{i}$ ← RandomSolution(n, G) (Algorithm 1)17:          **while** ${F(X}_{i})\;=\;0\;$**or** SN contains $X_{i}$18:          SN[i] ← $X_{i}$19:          ${trial}_{i}$ ← 020:     **end for**21:     ($X_{best},\;f_{best}$) ← UpdateBest(SN,$\;X_{best},\;f_{best}$)22:
**end for**


### 4.3. Binary Artificial Bee Colony (BABC) Adaptation

Standard ABC implementations operate in continuous domains using floating-point vector arithmetic. To adapt the collective foraging mechanism to the binary combinatorial space of parcel selection without continuous rounding, this study implements a Binary Artificial Bee Colony (BABC) formulation based on bitwise Boolean operators [20].

#### 4.3.1. Food Source and Nectar Representation Adaptation

- Nectar Representation: A binary bit where 1 indicates parcel selection for harvesting and 0 indicates exclusion [17].
- Food Source: A fixed-length binary vector (Equation (17)) of length |V| (total concession parcel count), its evaluated fitness value $F(X_{i})$, $count(X_{i})\;$ is the number of binary bits in 1 and a visit counter ${trial}_{i}$.

(17) $$X_{i}=\{p_{1},p_{2},\;...\;,p_{\left|V\right|}\}$$

- Colony Population and Search Boundaries: The colony manages a population of SN food sources whose cardinality (number of selected parcels) is bounded by lower and upper operational search boundaries, $L_{inf}$ and $L_{sup}$, respectively:

(18) $$count(L_{inf})\leq count(X_{i})\leq count(L_{sup})$$

- Boundary Violation Replacement and Food Source Exhaustion: The candidate evaluation, boundary restriction mechanism, and scout abandonment logic in BABC follow the exact same formulation described for Set-ABC in Section 4.2.4 (Algorithm 2). The only distinction lies in the neighborhood generation operator, where the candidate solution is constructed using the bitwise Boolean formulation (Equations (17) and (18)) instead of the set-theoretic perturbation mechanism (Equations (13) and (14)).

#### 4.3.2. Bitwise Operators and Neighborhood Search

Neighboring food source construction $V_{i}$ from an active food source $X_{i}$ and a randomly chosen food source $X_{k}$ is governed by bitwise Boolean logic rather than continuous vector arithmetic [20]. The bitwise operators are defined as follows:

1. Bitwise Exclusive OR (⊕): Given binary solutions A  and B, C = A⊕B identifies coordinate-level discrepancies between both configurations, returning 1 for indices where the parcel selection states differ and 0 where they match.
2. Bitwise AND (⨂): Given binary vectors A and B, C = A⨂B performs the standard conjunction operation across each bit position, yielding 1 if and only if the corresponding bits in both A and B are equal to 1, and 0 otherwise.

#### 4.3.3. New Food Source Construction Mechanism Adaptation

In contrast to the set-theoretic operators deployed in Set-ABC, the Binary Artificial Bee Colony (BABC) generates candidate solutions over a binary domain using bitwise Boolean arithmetic. Given the current binary food source $X_{i}\;\in \;{\left\{0,1\right\}}^{\left|V\right|}\;$and a distinct randomly selected neighbor $X_{k}\;\in \;{\left\{0,1\right\}}^{\left|V\right|}$, a candidate binary solution$\;V_{i}$ is constructed via [18]:

(19) $$V_{i}\;=\;X_{i}⊕(B\;⨂\;(X_{i}⊕X_{k}))$$

where:

○ ⊕ and ⨂ denote the bitwise XOR and bitwise AND operators defined in Section 4.3.2, respectively.
○ $B\;\in \;{\left\{0,1\right\}}^{\left|V\right|}$ is a stochastic binary vector of length units $\left|V\right|$ that replaces the continuous scaling factor ϕ. Each element $B_{j}$ is sampled independently from a Bernoulli distribution with equal probability (p=0.5):

(20) $$B_{j}=\left\{\begin{array}{c} 1,\;\;\;if\;U(0,1)\;>\;p\\ 0,\;\;otherwise \end{array}\right.$$

Here, the term in $\left(X_{i}⊕X_{k}\right)$ identifies the exact parcel selection discrepancies between the two solutions, while the conjunction with vector B selectively filters which discrepancies will perturb $X_{i}$. The final XOR operation applies these filtered changes, producing a new candidate binary configuration $V_{i}$ whose active parcel count is recalculated prior to fitness evaluation (Algorithm 5):
**Algorithm 5:** Pseudocode for the food source construction BABCInput: i (food source index), SN Output: $V_{i}$(new food source)1:k ← RandomIndex(0,|SN|)2:$X_{i}$ ← SN[i]3:$X_{k}$ ← SN[k]4:**if** i ≠ k **then**5:     B ← InitializeVector($\left|X_{i}\right|,$0)6:     **for each** $B_{j}$ **in** B7:            $B_{j}$ ← RandomRealNumber(0,1) > 0.58:     **end for**
9:     $V_{i}=X_{i}⊕(B\;⨂\;(X_{i}⊕X_{k}))$
10:**else**11:     $V_{i}$ ← $X_{i}$12:**end if**

#### 4.3.4. Source Replacement and Bounded Evaluation Adapted

The source evaluation and selection mechanisms in BABC mirror the logic formulated for Set-ABC in Section 4.2.4 (Algorithm 2). The primary operational difference lies in how candidate solution cardinality is computed: because solutions in BABC are encoded as fixed-length binary vectors rather than variable-sized sets, the set-cardinality operator $\left|V_{i}\right|$ is replaced by $count(V_{i})$, defined as the total number of active bits (1’s) across the vector.

This active-bit count represents the exact number of selected harvesting parcels. If the newly generated binary solution $V_{i}$ improves fitness over $X_{i}$. its active parcel count is checked against the search limits. If Equation (18) is false, the solution is reset to the corresponding boundary benchmark (${trial}_{i}$ ← ${trial}_{i}$+ 1).

#### 4.3.5. Overall Binary Artificial Bee Colony Algorithm

The overall execution flow of the Binary Artificial Bee Colony (BABC) algorithm follows the exact same structural framework as the Set-based ABC described in Section 4.2.5. It integrates the food source initialization, the three foraging phases (Employed, Onlooker, and Scout bees), and the global best solution memorization across iterations (Algorithm 6), differing only in the underlying representation—binary bit-vectors instead of parcel-identifier sets—and the corresponding discrete operators used for neighborhood construction.
**Algorithm 6:** Pseudocode for Binary Artificial Bee Colony (BABC)Input: MaxIter, Limit, Size, $L_{inf}$(lower limit), $L_{sup}$(upper limit) Output: $X_{best}$ (best food source),$\;f_{best}$ (best fitness)1:SN ← InitializePopulation($Size,L_{inf},\;L_{sup}$)2:**for** it = 1 to MaxIter **do**3:     **for** i = 1 to |SN| **do//**Employed phase4:          $X_{i}$ ← SN[i]5:          $V_{i}$ ← NewSourceConstruction(i,SN) (Algorithm 5)6:          SN[i] ← FoodSourceEvaluation($X_{i},V_{i},L_{inf},L_{sup}$) (Algorithm 3 using *count*)7:     **end for**
8:     **for** i = 1 to |SN| **do//**Onlooker phase9:          k ← SelectionFoodSourceIndex(“Roulette Wheel”,SN)10:          $V_{k}$ ← NewSourceConstruction(k,SN) (Algorithm 5)11:          SN[k] ← FoodSourceEvaluation($X_{k},V_{k},L_{inf},L_{sup}$) (Algorithm 3 using *count*)12:     **end for**
13:     **For each** $X_{i}$ **with** ${trial}_{i}\geq Limit\;$**do//**Scout phase14:          **do**15:                 n ← RandomPositiveNumber($count(L_{inf}),count(L_{sup})$)16:                 $X_{i}$ ← RandomSolution(n,G) (Algorithm 1)17:          **while** ${F(X}_{i})\;=\;0\;$**or** SN contains $X_{i}$18:          ${trial}_{i}$ ← 019:     **end for**20:     ($X_{best},\;f_{best}$) ← UpdateBest(SN,$\;X_{best},\;f_{best}$)21:**end for**

### 4.4. Genetic Algorithm (GA) Adaptation

To benchmark the performance of the set-theoretic ABC algorithm, a multi-point Genetic Algorithm (GA) is implemented using a traditional binary encoding scheme.

#### 4.4.1. Chromosome and Population Structure

- Gen Representation: A binary bit where 1 indicates parcel selection for harvesting and 0 indicates exclusion.
- Chromosome/Individual: A fixed-length binary vector (Equation (21)) of length |V| (total concession parcel count), paired with its calculated fitness $F(X_{i})$.

(21) $$X_{i}=\{p_{1},p_{2},\;...\;,p_{\left|V\right|}\}$$

- Population: A collection of individuals evolved over a fixed number of generations.

#### 4.4.2. Genetic Operators

- Parent Selection: Employs roulette wheel selection proportional to individual fitness, complemented by a replacement rate to preserve top-performing solutions across generations.
- Multi-Point Crossover: Swaps multiple sub-segments of binary chromosomes between two parents using randomly chosen cut points. Segments are exchanged based on the crossover rate (CRate) [12,14].
- Single-Point Bit-Flip Mutation: Operates hierarchically at two levels to introduce local genetic diversity:
  ○ Probability Mutation (Pmut): Operates at the gene level. For each selected offspring, a target locus is flipped (0 → 1 or 1 → 0) with probability Pmut, which corresponds to including or excluding an individual forest parcel from the harvest plan.
  ○ Mutation Rate (MRate): Governs the individual level, determining the proportion of offspring in the mating pool that is subjected to the mutation operator.
- Survivor Selection: Applies ranking-based replacement to construct the next generation, retaining high-fitness parents alongside newly mutated offspring [13].

#### 4.4.3. Overall Binary Genetic Algorithm

The overall execution flow of the adapted Binary Genetic Algorithm integrates five sequential phases executed across successive generations (Algorithm 7):

- Initialization: Generates an initial population of binary chromosomes of size (Size), where each bit randomly represents parcel selection.
- Parent Selection: Chooses candidate parent chromosomes through roulette wheel selection biased toward higher fitness values, retaining an elitist fraction defined by Replacement Rate (RRate).
- Parent Crossover: Combines pairs of selected parents via multi-point crossover according to CRate, interchanging chromosome segments to produce preliminary offspring.
- Offspring Mutation: Modifies a fraction MRate of generated children by flipping individual parcel bits according to probability Pmut to maintain genetic diversity and prevent premature convergence.
- Survivor Selection: Evaluates the candidate offspring, sorts individuals using ranking selection, and constructs the next generation by merging the best-performing parents with the highest-fitness offspring.

**Algorithm 7:** Pseudocode for Genetic Algorithm (GA)Input: MaxIter, Size, RRate (Replacement Rate), CRate (Crossover Rate), MRate (Mutation Rate), Pmut (Probability Mutation) Output: $X_{best}$ (best individual source),$\;f_{best}$ (best fitness)1:population ← InitializePopulation(Size)2:**for** it = 1 to MaxIter **do**3:     selectedParents ← ParentsSelection(“Roulette Wheel”, population, RRate)4:     newChildren ← ParentsCrossover(“Multi-Point Crossover”, selectedParents, CRate)5:     OffspringMutation(“Single-Point Bit-Flip Mutation”, newChildren, MRate, Pmut)6:     population ← SurvivorSelection(“Ranking”, population, newChildren, RRate)7:     ($X_{best},\;f_{best}$) ← UpdateBest(population,$\;X_{best},\;f_{best}$)8:**end for**

### 4.5. Simulated Annealing (SA) Adaptation

Simulated Annealing is implemented as a single-solution trajectory metaheuristic to explore whether a local search mechanism guided by probabilistic uphill transitions can effectively navigate the constrained parcel-selection landscape [11].

#### 4.5.1. Solution Representation and Initial State

A candidate solution X is represented as a dynamic list of parcel identifiers (Equation (22)):

(22) $$X=\;\{p_{1},p_{2},\;...\;,p_{k}\}$$

matching discrete set-based encoding utilized in Set-ABC. The search initiates from an initial state $X_{0}$ generated using Algorithm 8, guaranteeing that the trajectory starts from a regulation-compliant configuration with positive net profit F(X) > 0.
**Algorithm 8:** Pseudocode for Construct Feasible SolutionInput: G (Graph of parcels) Output: X (Solution with F(X) > 0)1:  **do**2:         X ← RandomSolution($\left|G.V\right|,G$) (Algorithm 1)3:  **while** F(X) = 04:($X_{best},\;f_{best}$) ← (X, F(X))5:**end for**

#### 4.5.2. Neighborhood Generation Strategy: Single-Element Variation

Candidate neighbors$\;X\in N(X_{0})$ are produced via the *Single-Element Variation* operator (Algorithm 9). At each step, a candidate copy is perturbed by either expanding or pruning the current harvest block, governed by a stochastic decision threshold $\left(p_{decision}\;\in \;[0,1]\right)$:

- Parcel Addition ($random(0,1)\;<p_{decision}$): The operator queries the concession adjacency matrix to gather all unprotected candidate units bordering the active block. After shuffling this candidate neighborhood, adjacent parcels are sequentially tested by temporarily inserting them into the block and evaluating feasibility. The first neighbor yielding a feasible configuration (or upon reaching the search cap of tested candidates) is retained.
- Parcel Removal ($random(0,1)\;\geq p_{decision}$): The operator randomly shuffles the selected parcels in the current solution and tests the removal of individual parcels one by one. The first deletion that preserves block feasibility (ensuring connectivity is not broken and enclave limits are respected) is committed.

If no feasible single-element modification is found within the candidate exploration limit, the perturbed state is evaluated as generated, triggering the death-penalty assignment (F(X) = 0) if constraints are breached.
**Algorithm 9:** Pseudocode for Single-Element Variation Neighborhood GenerationInput: $X_{0}$ (Current solution), $p_{decision},$ max_eval, (maximum neighbor attemps, set to 1), G (Graph of parcels) Output: X(Solution with F(X) > 0)1:X ← clone($X_{0})$2:p ← RandomRealNumber(0,1)3:**if** $p<p_{decision}$ **then**4:    unprotected ← FilterUnprotectedParcels(G)5:    visited ← InitializeVisitedMap($X_{0},$ unprotected,True)6:    N ← GetNeighborhoodOf(X, G, visited)//function used in Algorithm 17:    Shuffle(N)8:    attempts ← 09:    **for each** neighbor **in**
N10:          Add(X, neighbor)11:          **if** F(X) > 0 **or** attempts > max_eval **then**12:              **break**13:          **end if**14:          Remove(X, neighbor)15:          attempts ← attempts + 116:    **end for**17:**else**18:    selected_parcels ← extract(X)19:    attempts ← 020:    **for each** parcel **in** selected_parcels21:          Add(X, parcel)22:          **if** F(X) > 0 **or** attempts > max_eval **then**23:          Remove(X, parcel)24:          attempts ← attempts + 125:    **end for**26:**end if**

#### 4.5.3. Acceptance Criterion, Cooling Schedule, and Algorithmic Workflow

Because the objective function calculates net economic profit in monetary units (PEN), raw fitness deviations between states can vary by multiple orders of magnitude. Evaluating unscaled differences would instantly saturate the exponential acceptance term. Consequently, relative transitions are evaluated through a normalized objective difference $\delta_{norm}$ scaled against the current state fitness (Equation (23)):

(23) $$\delta_{norm}=\;\frac{F(X_{0})\;-\;F(X)}{F(X_{0})}$$

For a maximization framework:

- If ($\delta_{norm}\;<\;0$): the candidate neighbor yields higher net profit and is unconditionally accepted ($X_{0}\;\leftarrow \;X$).
- If ($\delta_{norm}\;\geq \;0$): the deteriorating transition is accepted probabilistically according to the Metropolis criterion (Equation (24)):

(24) $$P(accept)\;=\;e^{\frac{-\delta_{norm}}{T}}$$

where a random value x ∈ [0,1] is sampled; if $x\;<\;e^{\frac{-\delta_{norm}}{T}}$, the worsening move is committed to allow escaping local optima.

System temperature decays geometrically according to $T_{k+1}\;=\;\alpha T_{k}\;$where α ∈ ]0,1[ denotes the cooling coefficient applied after running an equilibrium chain of iterations ITER at each temperature stage. The entire optimization sequence is detailed in Algorithm 10.
**Algorithm 10:** Pseudocode for Simulated Anneling (SA)Input: $T_{0}$ (Initial Temperature), $T_{min}$ (Stopping Temperature), α (Cooling rate), ITER (iterations per temperature level), $p_{decision}$ (Decision Probability), max_eval (Maximum number of attempts) Output: $X_{best}$ (best food source),$\;f_{best}$ (best fitness)1:$X_{0}$ ← ConstructFeasibleSolution(G) (Algorithm 8)2:T ← $T_{0}$3:($X_{best},\;f_{best}$) ← ($X_{0},\;F(X_{0})$)4:**while** $T>T_{min}$ **do**5:     **for** iter = 1 **to** ITER **do**6:          X ← GenerateSingleChangeNeighbor($X_{0},p_{decision},max\_eval,G$) (Algorithm 9)7:          **if** F(X)=0 **then**
8:               X ← ConstructFeasibleSolution(G) (Algorithm 8)9:          **end if**10:          $\delta_{norm}=\frac{F(X_{0})-F(X)}{F(X_{0})}$
11:          **if** $\delta_{norm}>0$ **then**
12:               $X_{0}$ ← X13:               ($X_{best},\;f_{best}$) ← UpdateBest($X_{0},$$\;X_{best},\;f_{best}$)14:          **else if** randomRealNumber(0,1) $<\;e^{\frac{-\delta_{norm}}{T}}$**then**15:                 $X_{0}$ ← X16:          **end if**17:     **end for**18:     T← αT
19:**end while**

## 5. Implementation and Experimental Setup

This section outlines the software stack, execution environment, synthetic dataset specifications, hyperparameter tuning protocol, and statistical comparison pipeline used to evaluate both metaheuristics.

### 5.1. Software Architecture and Execution Environment

The optimization platform was developed in Java 21 using the Spring Boot framework (v4.0.3) for service orchestration and experiment automation. Automated testing and unit validation were implemented using the Spring Boot Starter Test module, incorporating JUnit 5 and Mockito frameworks. Experiments were executed on an 8-core x86_64 architecture running 64-bit Windows 10 Pro, powered by an Intel Core i5-10310U CPU @ 1.70 GHz (2.21 GHz boost) with 16.0 GB of installed RAM.

### 5.2. Dataset and Instance Generation

Algorithm performance was benchmarked on a semi-synthetic dataset derived from the real operational forest inventory of the ecological plantation “EL PAISA” located in Nauta, Loreto, Peru. The concession landscape discretizes a total surface area ($S_{total}$) of approximately 99 hectares into |V|≈ 90 contiguous land parcels. The instance incorporates 12 commercial Amazonian timber species identified across the operational inventory [12].

To construct a realistic yet controlled benchmark under the updated regulatory and economic formulation, data parameters were integrated and synthesized from multiple official and experimental sources:

- Spatial Parcel Topography and Floristic Inventory**:** Baseline tree species occurrences, diameter classes, and spatial distributions were drawn from the primary forest census at “EL PAISA”. To adapt these counts to parcel-level management units, tree densities, species counts per parcel, and canopy coverage fractions were generated using bounded uniform sampling calibrated against the operational field density ranges.
- Statutory and Conservation Baselines: Minimum forest canopy cover ($R_{CC}=\;0.25$), native primary forest conservation ratios ($R_{FC}\;=\;0.3$), and protected exclusion zones were anchored in national forestry thresholds mandated by Supreme Decree No. 018-2015-MINAGRI [24]. Species-specific conservation ratios reflect official categorization lists and CITES-Peru flora classifications [4].
- Economic Valuation, Extraction Costs, and Penalties: Commercial wood sale values per cubic meter were established using regional timber market references from SERFOR [5]. Species-level extraction costs and infraction penalty coefficients were derived from the administrative valuation methodology formulated in Presidential Resolution No. 021-2018-OSINFOR (*Metodología N.° 001-2018-OSINFOR*) [25].
- Experimental Parameters: Operational limits such as the available project execution budget, timber volume conversions, and the internal enclave tolerance ceiling were configured experimentally to provide a balanced, non-trivial combinatorial search space. Furthermore, candidate parcel-search boundaries governing solution initialization and replacement bounds in the Artificial Bee Colony variants (Set-ABC and BABC)—spanning exploration windows from 25% to 80% of the total dataset parcels $\left|V\right|$ are fully documented and accessible within the public project repository.
- The global problem parameters are summarized as follows:
- Available Project Budget ($B_{proj}$): 900,000 PEN.
- Minimum Forest Canopy Coverage Ratio ($R_{CC}$): 25% (0.25).
- Primary Forest Conservation Ratio ($R_{FC}$): 30% (0.30).
- Maximum Internal Enclave Tolerance ($N_{u}$): 5 unharvested internal parcels.

### 5.3. Hyperparameter Calibration

To guarantee a rigorous and computationally fair comparison, algorithm hyperparameters were calibrated using a systematic single-round Grid Search (GS) over the semi-synthetic forest instance. Unlike multi-round or asymmetric heuristics, all population-based metaheuristics (Discrete Set-based ABC, Binary ABC, and Binary GA) operated under a common computational budget fixed at 1000 iterations (or generations) and a population size of 300 individuals (or food sources) [14,26].

For Simulated Annealing (SA), the geometric cooling schedule $T_{k}\;=\;T_{0}\alpha^{k}$ was analytically calibrated to match this computational budget. The initial temperature was set to$\;T_{0}=\;-0.25/\ln\left(0.9\right)\approx \;2.3728$ and the minimum (stopping) temperature to $T_{min}=\;-0.001/\ln\left(0.0001\right)\;\approx \;1.0857\times 10^{-4}$. Under a cooling rate α = 0.99, the outer temperature loop yields:

(25) $$K=\;\frac{\ln\left(T_{min}/T_{0}\right)}{\ln\left(\alpha \right)}=995\approx 1000\;steps$$

To align SA with the 300 evaluations per iteration of the population-based methods, the internal search budget was factored into combinations satisfying Iterations per temperature level (ITER) times Neighborhood candidates (max_eval), ensuring an identical evaluation budget across all four algorithms.

The structural baseline parameters held constant across the calibration are detailed in Table 1.

The discrete parameter grid evaluated candidate operators governing exploration-exploitation trade-offs, neighborhood perturbation windows, and selection pressures. The complete grid of tested candidate values is summarized in Table 2.

The optimal configuration for each metaheuristic was determined through a single-round Grid Search evaluated across four representative test seeds (42, 640, 1000, and 2000). The hyperparameter selection followed a modal-frequency criterion: the configuration most frequently identified as optimal across the four seeds was selected as the final setting. In the event of a tie in selection frequency between candidate configurations, the tie was resolved in favor of the parameter set that achieved the strictly higher net economic return (fitness) in Table 3.

The final selected hyperparameter configuration (Table 4):

○ Genetic Algorithm (GA): The parameter configuration CRate = 0.5, MRate = 0.5, Pmut = 0.9, RRate = 0.7 emerged as the modal setting across test seeds (selected in 50% of the trials, seeds 42 and 640). This parameter consistently reached the maximum global net return of 1,061,032.19 PEN prior to generation 300, demonstrating rapid convergence and high search stability.
○ Set-based ABC: The configuration characterized by an abandonment limit of Limit = 30, parcel search boundaries bounded between [25%, 75%] of |V|, and roulette-wheel selection was the most frequently selected setup across seeds (seeds 640 and 2000). Furthermore, this setting attained the highest overall net economic return recorded for the set-theoretic approach (986,147.00 PEN).
○ Binary ABC (BABC): The parameter set combining Limit = 40, search boundaries bounded between [50%, 80%] of |V|, and roulette-wheel selection was selected in half of the tuning runs (seeds 640 and 2000). However, when compared with the combinations of Limit = 30 (seeds 1000 and 42), the [50%, 80%] threshold consolidated a presence in 3 of the 4 seeds evaluated.
○ Simulated Annealing (SA): The cooling and neighborhood exploration scheme of 20 inner iterations per temperature with 15 candidate neighbors per iteration proved dominant, appearing in three of the four tuning seeds (42, 1000, and 2000). To resolve the tie between decision constants 0.50 and 0.60, the parameter 0.60 was chosen as it achieved the strictly superior net return on seed 1000 (1,041,478.61 PEN versus 1,039,281.63 PEN).

**Table 4:** Final configuration.

Algorithm	Best Parameters	Best Fitness
SA	Iterations per temperature	20
	Neighborhood sample size	15
	Decision constant (delta)	0.60
	Initial temperature (T0)	−0.25/ln(0.9) (~2.373)
	Minimum temperature (Tmin)	−0.001/ln(0.0001) (~1.086 × 10^−4^)
	Cooling rate (alpha)	0.99
BABC	Iterations	1000
	Population (Food sources)	300
	Abandonment limit (Limit)	30
	Search boundaries	[50%, 80%] of |V|
	Selection operator	Roulette Wheel
Set-ABC	Iterations	1000
	Population (Food sources)	300
	Abandonment limit (Limit)	30
	Search boundaries	[25%, 75%] of |V|
	Selection operator	Roulette Wheel
GA	Generations (Iterations)	1000
	Population size	300
	Parent selection	Roulette Wheel
	Crossover operator	Multi-Point Crossover
	Crossover rate (CRate)	0.50
	Type Mutation	Single-point bit-flip
	Mutation rate (MRate)	0.5
	Mutation probability (Pmut)	0.90
	Survivor selection	Ranking
	Replacement rate (RRate)	0.70

### 5.4. Comparative Protocol and Statistical Pipeline

To systematically evaluate algorithmic performance, stability, and convergence behavior under identical stochastic baselines, all four metaheuristics (Set-based ABC, Binary ABC, Binary GA, and Simulated Annealing) were executed over 20 independent runs across a shared set of 20 random numerical seeds. Because each algorithm was evaluated on the exact same sequence of pseudo-random seeds, the resulting fitness distributions constitute dependent (paired) samples across treatments [27].

Statistical validation followed an inferential decision tree structured to verify distributional assumptions before executing omnibus hypothesis testing and post hoc pairwise procedures:

1. Normality Assessment: The Shapiro–Wilk test was independently applied to each 20-run sample distribution at a significance threshold of (α = 0.05) to assess parametric normality across all four algorithms.
2. Variance Homogeneity Test: Levene’s test was conducted simultaneously across all four groups (α = 0.05) to verify the assumption of homoscedasticity.
3. Omnibus Hypothesis Testing:
  - If all four sample groups satisfied normality (p > 0.05) and the joint variance was homoscedastic (p > 0.05), a repeated-measures one-way Analysis of Variance (RM-ANOVA) was scheduled to test the global null hypothesis of equal algorithm means ($H_{0}:\;\mu_{SetABC}\;=\;\mu_{GA}\;=\;\mu_{BABC}\;=\;\mu_{SA}$).
  - If parametric normality was violated by any algorithm or if heteroscedasticity was present, the non-parametric Friedman test for paired samples was scheduled as the primary omnibus rank test ($\;H_{0}:\;{Median}_{SetABC}\;=\;{Median}_{GA}\;=\;{Median}_{BABC}\;=\;{Median}_{SA}$).
4. Post hoc Pairwise Comparisons: If the global omnibus test rejected $H_{0}(p<\;0.05)$, post hoc pairwise comparisons were conducted using the two-sided Wilcoxon signed-rank test for matched pairs across all six algorithm combinations (k = 6). To strictly preserve the family-wise error rate (FWER) and prevent Type I error inflation, the Bonferroni correction was applied, yielding an adjusted significance threshold of (Equation (26)):

(26) $$\alpha^{\prime }=\frac{\alpha }{k}=\frac{0.05}{6}\approx 0.0083$$

Differences satisfying ($p\;<\;\alpha^{\prime }$) were deemed statistically significant.

## 6. Results

This section presents the empirical performance comparison among the proposed Discrete Set-based Artificial Bee Colony (Set-ABC) algorithm, Binary Artificial Bee Colony (BABC), Binary Genetic Algorithm (GA), and Simulated Annealing (SA) when solving the parcel-based tree selection problem for sustainable Amazonian harvesting. To ensure a fair and rigorous comparative evaluation, all four metaheuristics were evaluated under an equivalent computational budget across 20 independent runs executed with a shared set of 20 pseudo-random seeds. The resulting best net economic return values (Equation (7)) obtained per run are reported in Table 5, providing the empirical baseline for the descriptive and inferential statistical pipeline.

To determine the appropriate inferential statistical pipeline (parametric Repeated-Measures ANOVA vs. non-parametric Friedman test) as outlined in Section 5.4, descriptive statistics were compiled, and formal distributional assumption tests were executed across all four treatment groups. Table 6 synthesizes the central tendency metrics, dispersion properties, parametric normality evaluations, variance homogeneity assessments, and the omnibus hypothesis test result.

- Assessment of Normality: The Shapiro–Wilk test indicated that the fitness distributions for SA (*p* = 0.8392), BABC (*p* = 0.0682), and the proposed Set-based ABC (*p* = 0.6013) did not significantly depart from parametric normality at the α = 0.05 threshold. In contrast, GA exhibited a severe violation of the normality assumption (W = 0.7210, *p* = $0.71135\;\times \;10^{-5}$), caused by its steep concentration and acute rightward kurtosis around the upper fitness boundary of 1,061,032.19 PEN.
- Assessment of Variance Homogeneity: Levene’s test confirmed pronounced heteroscedasticity across the four metaheuristics (Stat = 21.0248, *p* = $5.1164\;\times \;10^{-10}$ < 0.05). Simulated Annealing displayed the highest dispersion (SD = 14,205.60 PEN), reflecting the trajectory sensitivity inherent to single-solution annealing schedules, whereas the population-based methods achieved much tighter distributions, with GA reaching the lowest standard deviation (SD = 2315.86 PEN).
- Inferential Hypothesis Testing: Because the parametric assumptions of normality and homoscedasticity were both violated, the parametric RM-ANOVA was ruled out. Instead, the non-parametric Friedman test for paired samples across the 20 matched random seeds was conducted. The test yielded Chi-square = 56.16 with *p* = $3.8833\;\times \;10^{-12}$ < 0.05, decisively rejecting the null hypothesis of equal median algorithmic performance.

Following the rejection of the omnibus null hypothesis by the Friedman test, pairwise post hoc comparisons were conducted using the two-sided Wilcoxon signed-rank test for paired samples across the 20 matched random seeds. To control the family-wise error rate across all six pairwise combinations (k = 6), the Bonferroni adjustment established a corrected significance threshold of 0.0083 (Equation (26)). As summarized in Table 7, all pairwise comparisons yielded *p*-values strictly below $\alpha^{\prime }$, confirming statistically significant performance differences among all four metaheuristics. Specifically, GA significantly outperformed all candidate algorithms (*p* < $10^{-4}$), while Binary ABC attained a statistically significant advantage over both Simulated Annealing (*p* = $4.8256\;\times \;10^{-4}$) and the proposed Set-based ABC (*p* = $1.9073\;\times \;10^{-6}$).

To visually examine the structural performance hierarchy, distributional spread, and convergence stability across all four metaheuristics, Figure 1 illustrates the comparative boxplots paired with overlaid jittered individual runs across the 20 independent trials. The visualization confirms a clear, separated progression in median net economic return: the proposed Set-based ABC is centered at 983,046.01 PEN, followed by Simulated Annealing at 1,011,677.27 PEN, Binary ABC at 1,028,228.12 PEN, and GA at 1,060,753.26 PEN. Beyond median performance, the plot highlights distinct dispersion behaviors: Simulated Annealing exhibits the widest interquartile range (IQR = 20,884.72$ PEN) due to trajectory-dependent thermal exploration, whereas Binary GA displays extreme distributional concentration (IQR = 2154.19 PEN) around its top-performing state. Furthermore, while Binary ABC achieves higher median returns than Set-based ABC, the proposed discrete set formulation maintains a remarkably stable and compact variance profile (SD = 2823.15 PEN), demonstrating consistent convergence across independent stochastic evaluations.

To evaluate search efficiency, local optima avoidance, and convergence trajectories under an equivalent computational budget, the evolution of both local-best and cumulative global-best solutions was monitored across 1000 iterations for each method. The individual convergence profiles exhibit distinct exploratory behaviors as can be seen in Figure 2:

- Genetic Algorithm (GA): Characterized by an aggressive early ascent during the initial 50 generations, maintaining steady genetic refinement until achieving its definitive fitness plateau near generation 290. Beyond this stage, the iteration-best solution closely tracks the cumulative best-so-far trajectory, indicating that the elitist replacement and survivor ranking mechanisms drove the population into a stable, high-yield consensus attractor without premature stagnation.

**Figure 2:**
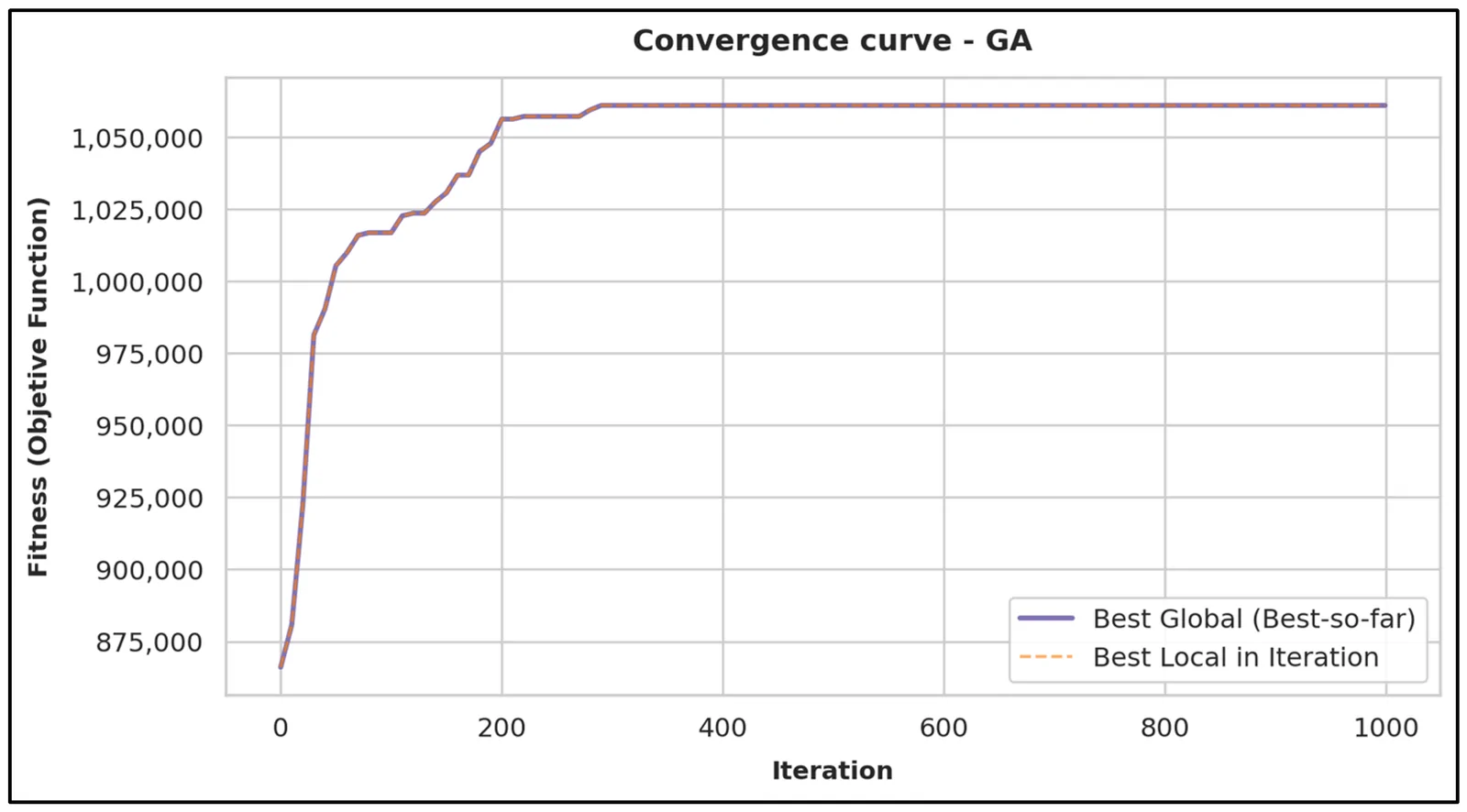
Convergence Curve GA best parameters showing iterations for each multiple of 10.

- Binary Artificial Bee Colony (BABC): Demonstrates rapid initial exploitation across the first 40 iterations, followed by stepped improvements resulting from scout-bee perturbations. The local iteration-best trajectory displays controlled oscillations below the cumulative curve, confirming that employed and onlooker bees maintained active neighborhood sampling while retaining the global best-so-far solution.
- Set-based Artificial Bee Colony (Set-ABC): Converges rapidly toward its primary plateau around iteration 100, with a final localized adjustment near iteration 470. Throughout the search, moderate local fitness fluctuations reflect continuous exploratory shuffling via set union ⊕ and difference ⊖ operators; however, these discrete perturbations stabilize within a lower-fitness basin compared to the binary representations.
- Simulated Annealing (SA): Exhibits severe variance and abrupt oscillations in the local iteration-best curve during the first 600 outer cooling cycles. This behavior is a direct consequence of high-temperature Metropolis–Hastings acceptance transitions that facilitate escape from local optima by accepting deteriorating moves. As temperature decays below critical thresholds, the trajectory gradually stabilizes, consolidating its final harvest block near iteration 720.

To directly compare learning velocity, escapability from deceptive local optima, and the final solution quality across methods, Figure 3, Figure 4, Figure 5 and Figure 6 synthesizes the cumulative best-so-far fitness trajectories over the 1000 iterations for all four evaluated metaheuristics respectively:

To address operational feasibility, constraint compliance, and practical forest management implications, the definitive harvest blocks identified by both the median and peak runs of each metaheuristic were analyzed across their spatial, economic, and biological dimensions. Table 8 details the parcel selection properties, harvested land and canopy distributions, economic returns, budget utilization, and legal retention ratios for the median solutions across the 90-parcel concession. Correspondingly, Table 9 provides the complete operational and ecological breakdown for the best overall solution discovered by each metaheuristic, establishing a direct baseline to evaluate management trade-offs under representative and peak algorithmic performance.

## 7. Discussion

The four-algorithm comparison yields a clear and statistically robust performance hierarchy. The Friedman test rejected the null hypothesis of equal median performance (χ^2^ = 56.16, *p* = 3.88 × 10^−12^), and all six Bonferroni-corrected Wilcoxon signed-rank comparisons were significant at the adjusted threshold α′ = 0.0083. Ranked by mean net economic return, the ordering is GA (1,059,364 PEN) > BABC (1,028,861 PEN) > SA (1,013,023 PEN) > Set-ABC (983,873 PEN): GA significantly outperformed every competitor, BABC was significantly superior to both SA and the proposed Set-ABC, and SA in turn significantly exceeded Set-ABC. Two findings stand out immediately. First, a well-tuned Binary GA is the method to beat on this regulated parcel-selection instance. Second, and less comfortably for the encoding hypothesis of this work, within the ABC family the binary variant outperformed the proposed set-theoretic adaptation under a matched computational budget.

Central tendency, however, tells only part of the story; dispersion adds a complementary reading. GA was simultaneously the most accurate and the most stable method (SD = 2315.86 PEN), while the proposed Set-ABC, despite the lowest mean, achieved the second-lowest standard deviation (SD = 2823.15 PEN), converging tightly and reproducibly to a consistent basin. Simulated Annealing sat at the opposite extreme (SD = 14,205.60 PEN), the expected signature of single-solution trajectory sensitivity—yet its best individual run (1,042,909.59 PEN) exceeded BABC’s best (1,041,572.84 PEN), showing that high-temperature exploration occasionally reaches excellent solutions it cannot reliably reproduce. The practical implication is that the results describe a quality–stability–cost frontier rather than a single dominant choice: GA for reliable peak quality, Set-ABC for stable and novel discrete search, and SA for cheap but volatile exploration.

Interpreted in operational terms, the gaps are narrower than the ranking suggests. All four methods converged to feasible, regulation-compliant harvest blocks of comparable spatial footprint—61 of the 90 parcels (~67.8%), roughly 66–67 ha harvested, with total-canopy and primary-forest retention comfortably above the statutory thresholds (R_CC = 25%, R_FC = 30%). The ~7% mean gap separating GA from Set-ABC therefore does not correspond to a categorically different management plan, but to finer optimization of the harvested species and volume mix. Concretely, GA paired high gross revenue with the lowest ecological penalties of the panel (104,142 PEN), whereas Set-ABC’s deficit is concentrated on the gross-revenue side (1,998,574 PEN versus ~2,065,000 PEN for the other three methods), indicating that it settles on a less valuable—but still fully legal—species composition. Every method delivers an auditable, compliant harvest schedule; they differ mainly in how much legal value they extract from a spatially similar block.

Convergence behavior under the shared 1000-iteration budget was equally distinctive. GA ascended aggressively and reached its plateau by roughly generation 290; BABC exploited early and refined through scout-driven steps (convergence near iteration 312); Set-ABC reached its primary plateau by about iteration 100 with a late adjustment near iteration 470 but stabilized within a lower-fitness basin; and SA oscillated through approximately 600 cooling cycles before consolidating near iteration 720. Computationally, the ordering inverts the quality ranking: SA was the cheapest method (43–77 s), GA the most expensive (142–246 s), with the two ABC variants in between. GA thus purchases its accuracy with the highest objective-function-call count and the longest runtime—a trade-off that matters where evaluation is costly or where near-real-time replanning is required. This asymmetry in maturity is central to interpreting the notion of “superiority.” GA relies on a well-understood operator set—roulette-wheel selection, multi-point crossover, and bit-flip mutation—supported by an extensive tuning literature, and a single-round Grid Search over four seeds converged it to a stable, aggressive configuration. Set-ABC, by contrast, is a novel adaptation whose dynamics depend on parameters—the abandonment limit, the parcel-selection boundaries, and the discrete perturbation magnitude drawn from φ—for which no consolidated tuning heuristics exist in this domain, and it was calibrated over the same grid without encoding-specific priors. Its consistent convergence into a lower but stable basin is compatible with sub-calibration rather than an intrinsic performance ceiling: the set-algebraic operators reliably locate a compact family of feasible solutions, but the current boundary and abandonment settings appear to under-explore the higher-value region that BABC and GA reach. That the binary ABC outperformed the set-theoretic one under identical budgets points specifically to the encoding–operator interaction as the place where dedicated tuning is most likely to yield gains.

Two limitations bound the generality of these conclusions. First, the evaluation rests on a single semi-synthetic instance of approximately 90 parcels derived from one operational distribution (the “EL PAISA” ecological plantation, Nauta, Loreto); the relative ordering could shift on larger or structurally different concessions, where the broader exploratory profile of swarm intelligence might become advantageous against GA’s tendency to concentrate around a single attractor. Second, all runs were executed on modest commodity hardware (an Intel Core i5 mobile CPU with 16 GB RAM), and iteration caps were set partly by runtime tractability rather than by convergence alone; a larger computational budget could reshape the trajectories of both methods, and of SA and Set-ABC in particular, given their later and more erratic discovery iterations. These observations are consistent with the broader forest-optimization literature, in which a well-tuned GA remains a strong baseline for spatial harvest problems [12,13,14], while swarm-based methods are typically reported as competitive rather than dominant until problem-specific adaptations mature [15,16,17]. Read in this light, the present study contributes not a claim that set-theoretic ABC surpasses established methods, but a statistically rigorous four-way benchmark establishing that GA is the reference to beat, that the proposed Set-ABC is a stable, feasible, and genuinely novel contender whose principal current weakness is gross-value extraction under an unspecialized calibration, and that the binary-versus-set encoding contrast is itself an informative result motivating encoding-aware tuning as the most promising next step.

## 8. Conclusions

This study addressed sustainable tree selection in the Peruvian Amazon, formulated as a regulated NP-hard combinatorial optimization over forest parcels, and delivered four contributions. First, a formal model of the problem was established, separating hard constraints—spatial connectivity, enclave tolerance, exclusion zones, budget, canopy cover, and primary-forest conservation—from a penalized species-retention constraint, all anchored in Law No. 29763, the national CITES registry, and Supreme Decree No. 018-2015-MINAGRI. Second, an objective function expressed directly in net economic return (PEN) was proposed, combining gross timber revenue and extraction cost while discounting statutory infractions through a species-level penalty, so that only legally and operationally admissible harvest blocks attain positive fitness. Third, a novel set-theoretic discrete adaptation of the Artificial Bee Colony algorithm (Set-ABC) was developed, representing food sources as parcel-identifier sets and driving neighborhood search through set-algebraic operators rather than continuous or binary encodings. Fourth, Set-ABC was benchmarked against a Binary ABC, a Binary Genetic Algorithm, and Simulated Annealing under a common computational budget and a paired statistical pipeline (Friedman test with Bonferroni-corrected Wilcoxon post hoc comparisons).

The four-way comparison produced a statistically robust performance hierarchy. All pairwise differences were significant at the adjusted threshold, with mean net return ordered GA (1,059,364 PEN) > BABC (1,028,861 PEN) > SA (1,013,023 PEN) > Set-ABC (983,873 PEN). A well-tuned Binary GA is thus the reference method to beat on this regulated instance, achieving both the highest accuracy and the lowest dispersion. Within the ABC family, the binary variant outperformed the proposed set-theoretic adaptation under matched budgets—an informative encoding-versus-set contrast rather than a dismissal of the discrete formulation. Indeed, the operational analysis showed that all four methods converged to feasible, regulation-compliant harvest blocks of comparable spatial footprint (61 of 90 parcels, retention above statutory thresholds); Set-ABC’s deficit was concentrated in gross-value extraction, not in feasibility or stability, and it achieved the second-lowest variance of the panel, converging reproducibly to a compact solution basin. Its consistent settling into a lower but stable region is compatible with sub-calibration under an unspecialized parameter grid rather than an intrinsic performance ceiling.

Several avenues follow directly from these findings. The most immediate is encoding-aware calibration of the ABC family: because Set-ABC was tuned over the same grid as the binary methods without operator-specific priors, dedicated tuning of its abandonment limit, parcel-selection boundaries, and discrete perturbation magnitude is the change most likely to close the observed gap. A natural extension is a Multi-Objective ABC (MOABC) that returns a Pareto front over net return, harvested area, and conservation compliance instead of collapsing them into a single scalar, yielding decision-support output better matched to real management trade-offs. Replacing random initialization with a GRASP-style constructive procedure could seed every method with high-quality feasible blocks and further mitigate early-search infeasibility, while hybrid memetic schemes coupling population-based exploration with local search would broaden the competitive landscape. Finally, extending the evaluation beyond the current single 90-parcel semi-synthetic instance to larger and structurally diverse concessions—ideally validated against field inventory data—would test the scalability of all four methods and clarify whether the broader exploratory profile of swarm intelligence becomes advantageous at scale.

## Figures and Tables

**Figure 1:**
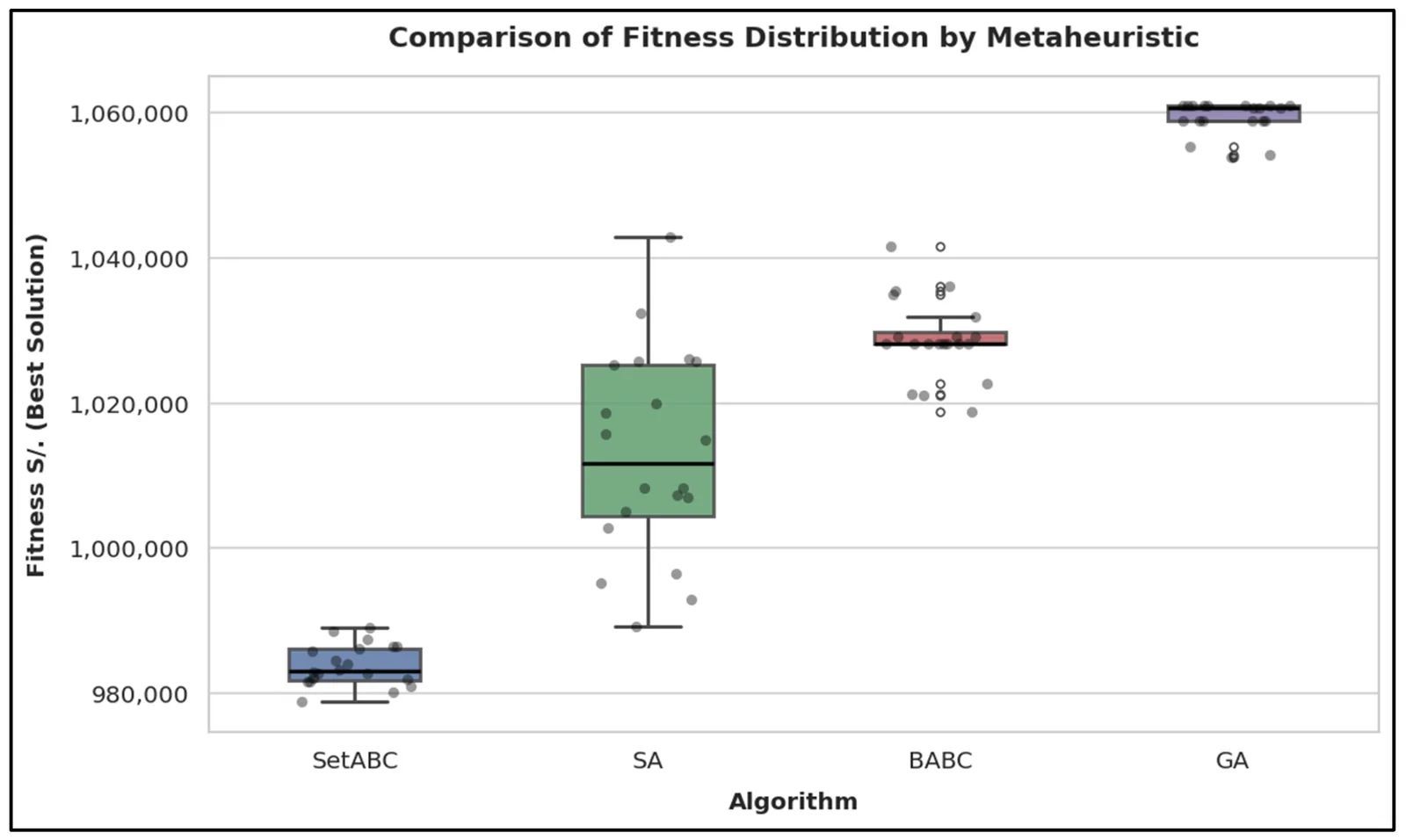
Boxplot comparing fitness distributions between the four metaheuristics across 20 independent runs.

**Figure 3:**
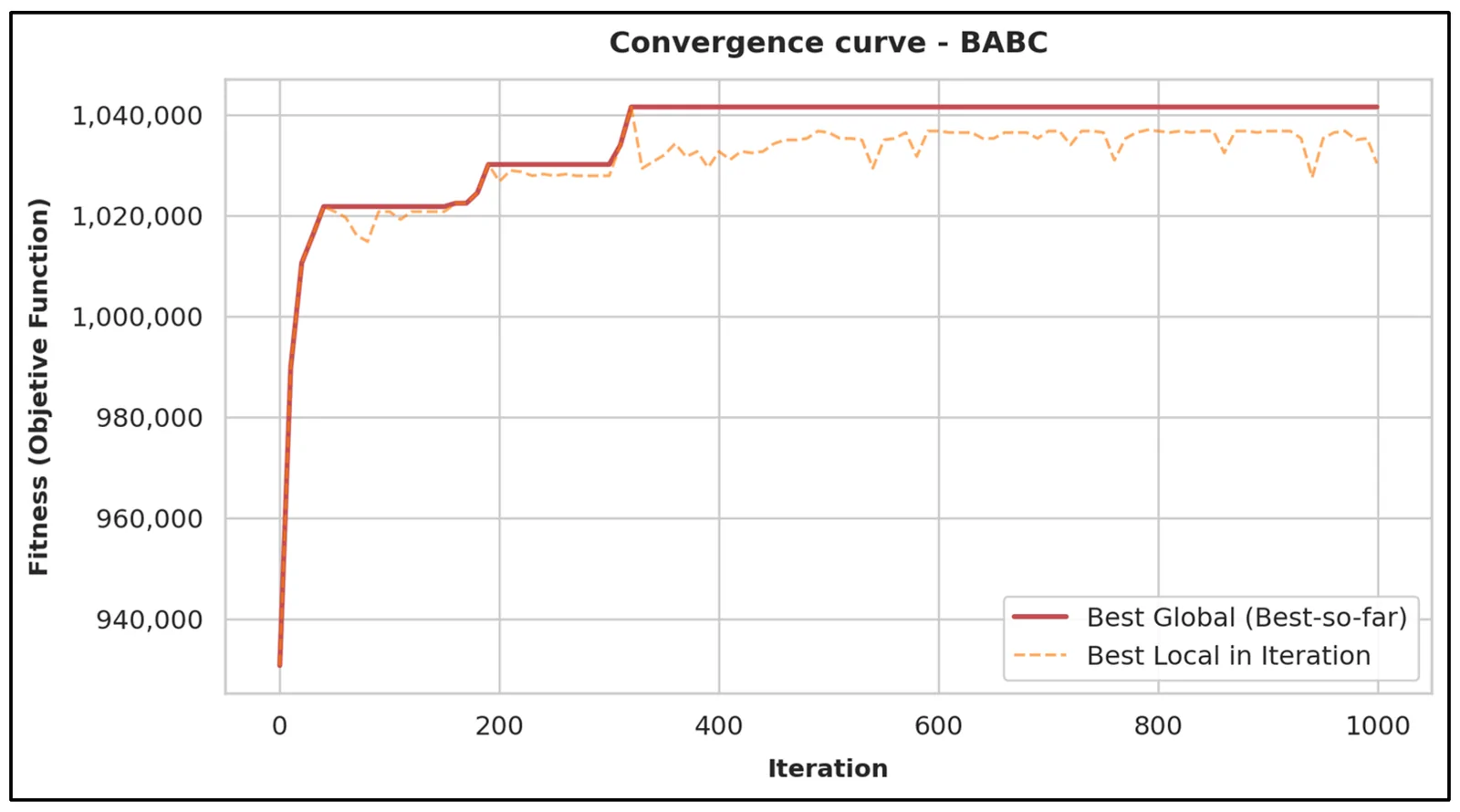
Convergence Curve BABC best parameters showing iterations for each multiple of 10.

**Figure 4:**
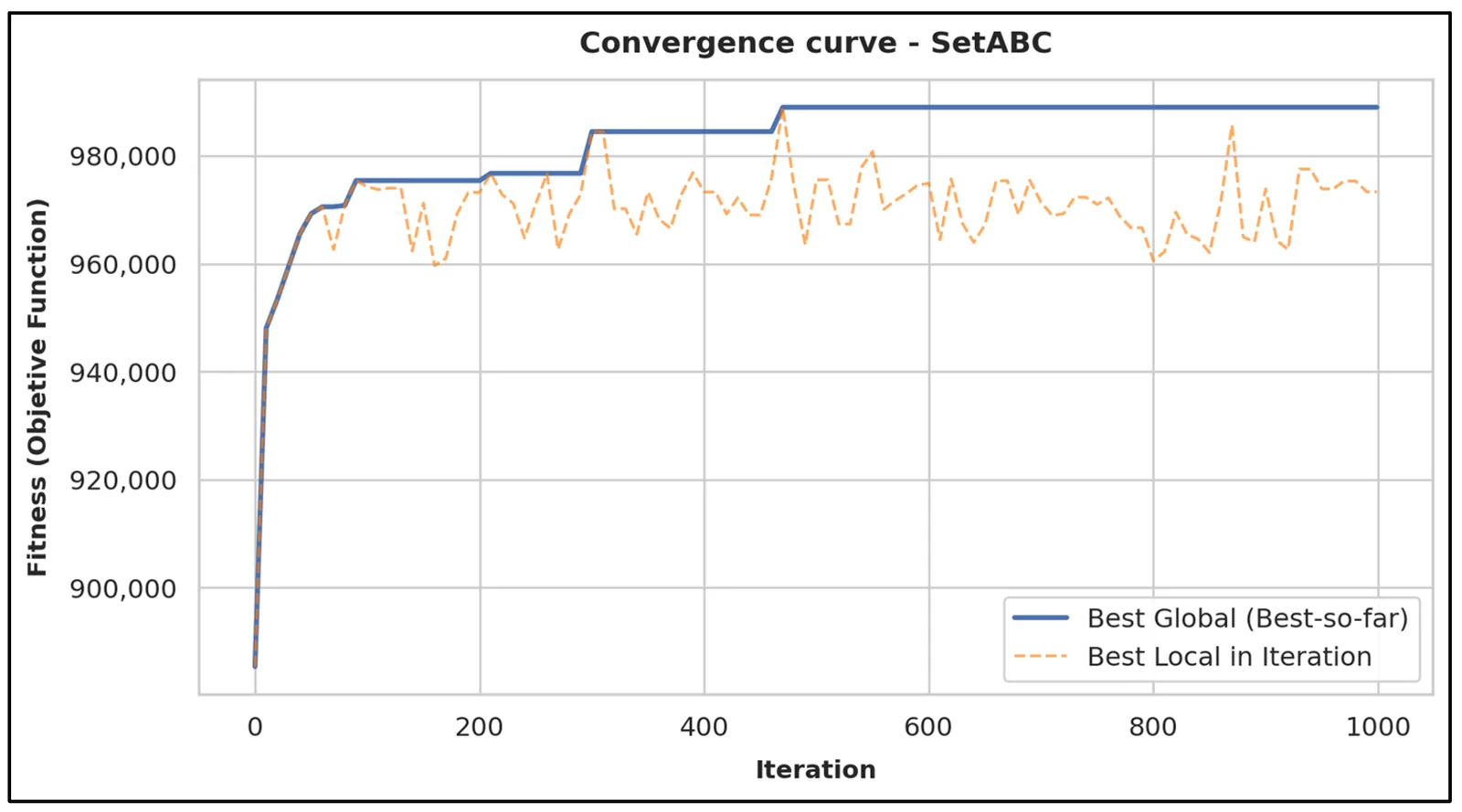
Convergence Curve SetABC best parameters showing iterations for each multiple of 10.

**Figure 5:**
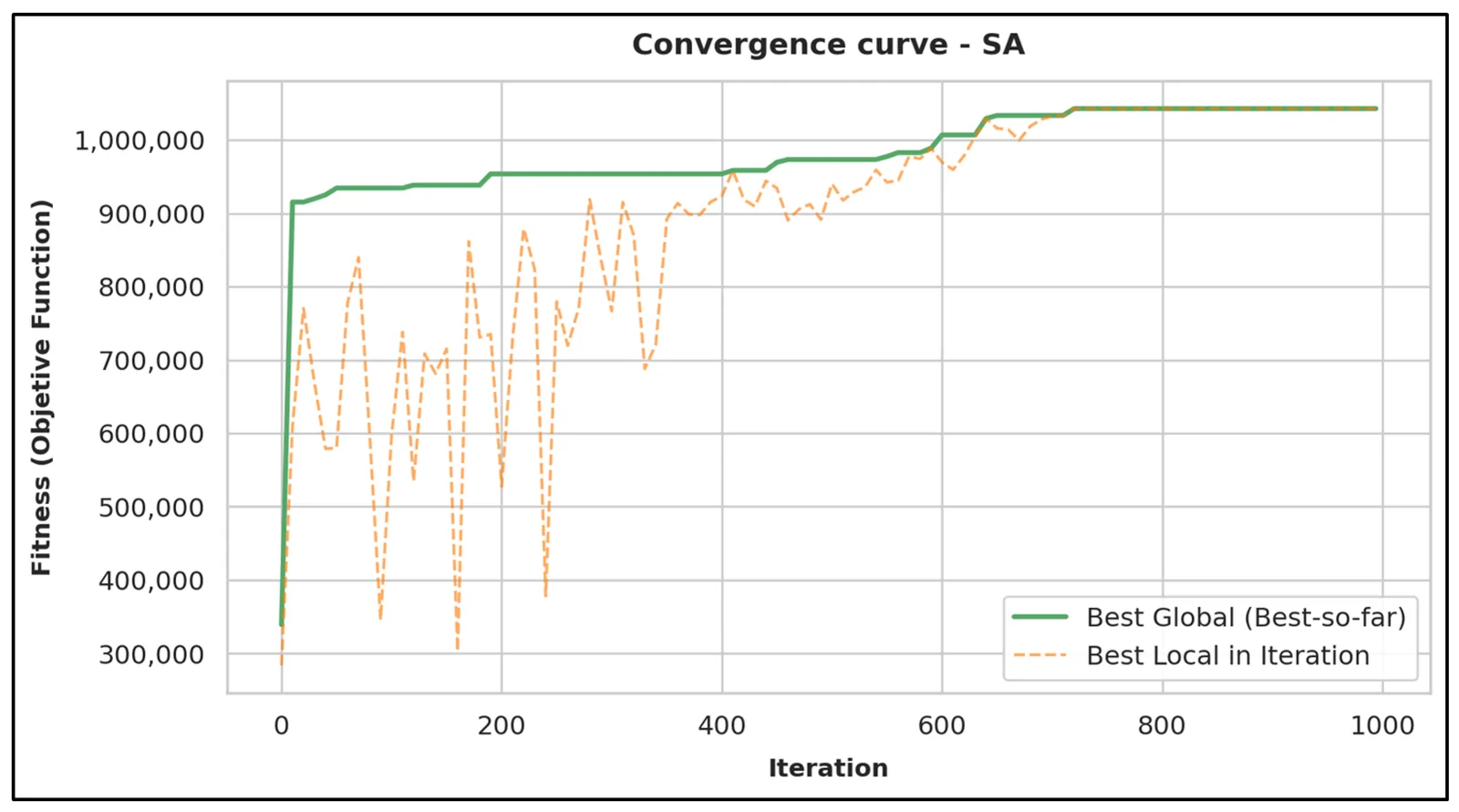
Convergence Curve SA best parameters showing iterations for each multiple of 10.

**Figure 6:**
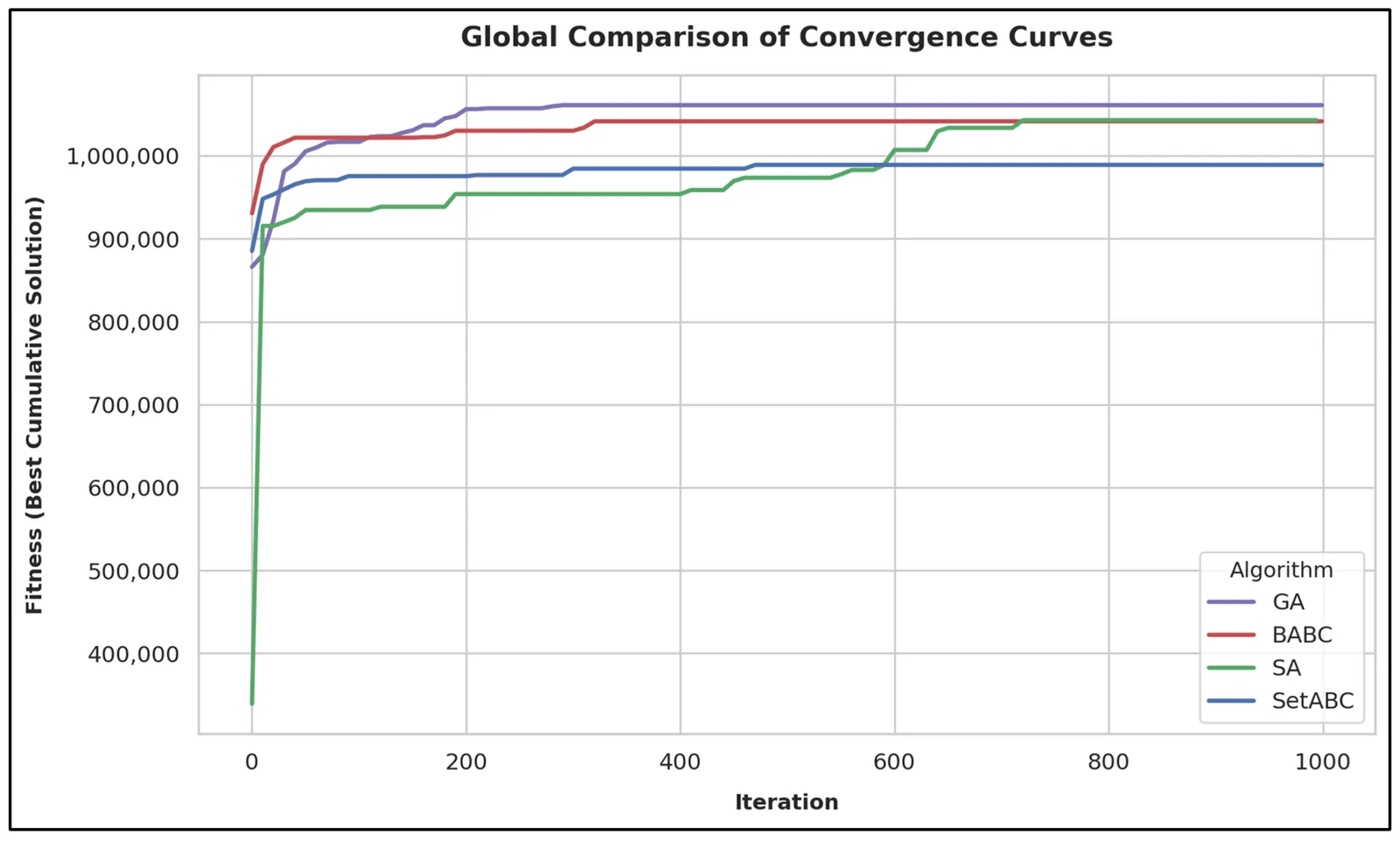
General comparisons.

**Table 1:** Baseline parameters across evaluated metaheuristics.

SA		BABC		SetABC		GA	
Name	Value	Name	Value	Name	Value	Name	Value
$T_{0}$	2.3728	Iteration	1000	Iteration	1000	Iteration	1000
$T_{min}$	$1.0857\times 10^{-4}$	Population	300	Population	300	Population	300
α	0.99	Constructor	Algorithm 1	Constructor	Equation (7)	Parent selection	Roulette wheel
-	-	Evaluation	Equation (3)	Evaluation	Equation (3)	Type crossover	Multi-point crossover
-	-	-	-	-	-	Type mutation	Single-point bit-flip
-	-	-	-	-	-	Survivor selection	Ranking

**Table 2:** Candidate hyperparameter search space tested in the Grid Search.

Algorithm	Hyperparameter	Value
SA	Iterations per temperature time Neighbors	(15,20); (20,15); (10,30)
	Decision constant	0.33, 0.40, 0.50, 0.60, 0.66
BABC	Abandonment limit	20, 30, 40
	Search boundaries	[30%, 70%], [25%, 75%], [50%, 80%] of |V|
	Food source selection	Roulette Wheel, Tournament (33% of |SN|), Tournament (25% of |SN|)
SetABC	Abandonment limit	20, 30, 40
	Search boundaries	[30%, 70%], [25%, 75%], [50%, 80%] of |V|
	Food source selection	Roulette Wheel, Tournament (33% of |SN|), Tournament (25% of |SN|)
GA	Crossover Rate	0.1, 0.3, 0.5
	Mutation Rate	0.1, 0.3, 0.5
	Probability Mutation	0.5, 0.7, 0.9
	Replacement Rate	0.5, 0.6, 0.7

Note: The GA parameters (Crossover Rate = 0.1, Mutation Rate = 0.1, Probability Mutation = 0.5, Replacement Rate = 0.6) were used by Gomide [14].

**Table 3:** Grid Search results.

Algorithm	Seed	Best Parameters	Best Fitness
BABC	42	Limit 30, [50%, 80%], Roulette	1,035,331.34
	640	Limit: 40, [50%, 80%], Roulette	1,042,712.98
	1000	Limit: 30, [30%, 70%], Roulette	1,044,223.15
	2000	Limit: 40, [50%, 80%], Roulette	1,030,204.39
Set-ABC	42	Limit: 30, [30%, 70%], Roulette	985,833.42
	640	Limit: 30, [25%, 75%], Roulette	986,147.00
	1000	Limit: 30, [50%, 80%], Roulette	983,385.56
	2000	Limit: 30, [25%, 75%], Roulette	986,147.00
GA	42	CRate = 0.5, MRate = 0.5, Pmut = 0.9, RRate = 0.7	1,061,032.19
	640	CRate = 0.5, MRate = 0.5, Pmut = 0.9, RRate = 0.7	1,061,032.19
	1000	CRate = 0.3, MRate = 0.5, Pmut = 0.9, RRate = 0.7	1,061,032.19
	2000	CRate = 0.5, MRate = 0.5, Pmut = 0.9, RRate = 0.6	1,061,032.19
SA	42	(20 iter./T × 15 neighbor), Decision = 0.50	1,039,281.63
	640	(15 iter./T × 20 neighbor), Decision = 0.50	1,037,601.93
	1000	(20 iter./T × 15 neighbor), Decision = 0.60	1,041,478.61
	2000	(20 iter./T × 15 neighbor), Decision = 0.60	1,036,141.17

**Table 5:** Best solution fitness values obtained over 20 independent algorithm runs.

Seed	SA	BABC	Set-ABC	GA
42	1,018,659.80	1,041,572.84	982,066.69	1,060,753.26
640	1,026,093.26	1,028,228.12	980,975.58	1,060,753.26
201	1,014,990.02	1,028,228.12	986,147.00	1,058,878.00
123	1,042,909.59	1,035,408.84	983,185.12	1,061,032.19
456	1,005,002.79	1,028,228.12	985,913.70	1,060,753.26
789	992,965.63	1,031,876.71	984,525.38	1,061,032.19
987	1,007,408.92	1,028,228.12	986,428.93	1,061,032.19
654	1,002,808.42	1,021,303.94	981,959.64	1,061,032.19
321	1,008,331.75	1,029,164.99	988,543.93	1,055,438.33
111	1,025,215.46	1,021,083.18	986,487.05	1,061,032.19
28	1,025,856.74	1,028,228.12	982,703.57	1,058,878.00
839	995,280.45	1,036,061.36	982,740.46	1,058,878.00
450	1,006,942.30	1,029,164.99	987,386.03	1,061,032.19
401	1,015,757.64	1,028,228.12	984,090.45	1,058,878.00
908	1,019,937.10	1,028,228.12	982,906.89	1,053,897.36
222	1,025,709.29	1,018,878.82	978,877.75	1,061,032.19
653	996,565.62	1,035,042.34	981,621.19	1,058,878.00
104	989,305.75	1,028,228.12	980,225.82	1,054,154.77
110	1,008,364.52	1,022,664.81	989,016.90	1,058,878.00
777	1,032,351.71	1,029,164.99	981,650.48	1,061,032.19

**Table 6:** Descriptive statistics and inferential test results across 20 independent runs.

Statistic/Test	SA	BABC	SetABC	GA
Mean Fitness	1,013,022.84	1,028,860.64	983,872.63	1,059,363.79
Median Fitness	1,011,677.27	1,028,228.12	983,046.01	1,060,753.26
Standard Deviation Fitness	14,205.60	5442.21	2823.15	2315.86
Max Fitness	1,042,909.59	1,041,572.84	989,016.90	1,061,032.19
Min Fitness	989,305.75	1,018,878.82	978,877.75	1,053,897.36
IQR Fitness	20,884.72	1614.80	4335.13	2154.19
Mean Run Time	77.32 s	179.51 s	203.35 s	246.22 s
Shapiro–Wilk Normality (*p*-value)	W = 0.9742 *p* = 0.8392 (Normal)	W = 0.9115 *p* = 0.0682 (Normal)	W = 0.9628 *p* = 0.6013 (Normal)	W = 0.7210, *p* = $7.1135\;\times \;10^{-6}$ (Non-Normal)
Levene’s Variance Test (*p*-value)	Stat = 21.0248, *p* = $5.1164\;\times \;10^{-10}$ (Heteroscedastic)			
RM-ANOVA	-			
Friedman test (χ^2^, *p*-value)	χ^2^ = 56.16 | *p* = $3.8833\;\times \;10^{-12}$ | reject $H_{0}$			

**Table 7:** Post hoc paired Wilcoxon signed-rank test results with Bonferroni correction.

Comparison Pair	Wilcoxon Statistic (W)	*p*-Value	Statistical Decision
SA vs. BABC	18.0	$4.8256\times 10^{-4}$	Statistically Significant
SA vs. Set-ABC	0.0	$1.9073\times 10^{-6}$	Statistically Significant
SA vs. GA	0.0	$1.9073\times 10^{-6}$	Statistically Significant
BABC vs. Set-ABC	0.0	$1.9073\times 10^{-6}$	Statistically Significant
BABC vs. GA	0.0	$8.7824\times 10^{-5}$	Statistically Significant
Set-ABC vs. GA	0.0	$1.9073\times 10^{-6}$	Statistically Significant

**Table 8:** Operational, ecological, and economic breakdown of the median solution found by each metaheuristic.

Operational/Ecological Metric	Simulated Annealing (SA)	Genetic Algorithm (GA)	Set-Based ABC (Set-ABC)	Binary ABC (BABC)	Threshold/Legal Boundary
Representative Random Seed	201	42	123	201	—
Selected Parcels (|X|/90)	60 (66.67%)	61 (67.78%)	62 (68.89%)	61 (67.78%)	Max 90 parcels
Harvested Land Area (ha/%)	65.06 ha (65.88%)	66.05 ha (66.88%)	67.82 ha (68.67%)	66.40 ha (67.24%)	Total: 98.75 ha
Harvested Canopy Area (ha/%)	47.17 ha (65.05%)	48.34 ha (66.65%)	49.63 ha (68.44%)	48.83 ha (67.34%)	Initial: 72.52 ha
Remaining Total Canopy Ratio	34.95% (Feasible)	33.35% (Feasible)	31.56% (Feasible)	32.66% (Feasible)	Mandatory RCC >= 25.00%
Harvested Primary Canopy Area (ha/%)	39.24 ha (65.65%)	40.95 ha (68.51%)	41.70 ha (69.76%)	39.81 ha (66.61%)	Initial: 59.77 ha
Remaining Primary Forest Ratio	34.35% (Feasible)	31.49% (Feasible)	30.24% (Feasible)	33.39% (Feasible)	Mandatory RFC >= 30.00%
Gross Timber Revenue (PEN)	2,047,656.29	2,055,509.93	2,023,832.06	2,033,654.86	Max: 3,099,712.93 PEN
Total Extraction Cost (PEN)	896,764.50	899,382.80	897,095.93	894,985.49	Max: 900,000.00 PEN
Budget Utilization (%)	99.64%	99.93%	99.68%	99.44%	Compliant (Phard = 0)
Economic Excess Penalties (PEN)	135,901.26	95,373.87	143,551.01	110,441.25	Soft ecological penalty
Net Economic Return (F(X) in PEN)	1,014,990.02	1,060,753.26	983,185.12	1,028,228.12	Objective function
Restricted Species Limit Violations	Palo Sangre (+42), Aguano Pashaco (+59)	Palo Sangre (+31), Aguano Pashaco (+49)	Palo Sangre (+52), Aguano Pashaco (+45)	Palo Sangre (+37), Aguano Pashaco (+49)	17 preserved, 2 penalized
Hard Ecological Constraints Status	Feasible (Phard = 0)	Feasible (Phard = 0)	Feasible (Phard = 0)	Feasible (Phard = 0)	100% legal compliance
Convergence Iteration/Budget	744/1000	345/1000	958/1000	89/1000	1000 iterations

**Table 9:** Operational, ecological, and economic breakdown of the best solution found by each metaheuristic.

Operational/Ecological Metric	Simulated Annealing (SA)	Genetic Algorithm (GA)	Set-Based ABC (Set-ABC)	Binary ABC (BABC)	Threshold/Legal Boundary
Representative Random Seed	123	789	110	42	—
Selected Parcels (|X|/90)	61 (67.78%)	61 (67.78%)	61 (67.78%)	61 (67.78%)	Max 90 parcels
Harvested Land Area (ha/%)	67.27 ha (68.12%)	65.95 ha (66.78%)	66.76 ha (67.60%)	66.01 ha (66.84%)	Total: 98.75 ha
Harvested Canopy Area (ha/%)	49.49 ha (68.24%)	48.29 ha (66.59%)	48.67 ha (67.11%)	48.44 ha (66.79%)	Initial: 72.52 ha
Remaining Total Canopy Ratio	31.76% (Feasible)	33.41% (Feasible)	32.89% (Feasible)	33.21% (Feasible)	Mandatory RCC >= 25.00%
Harvested Primary Canopy Area (ha/%)	40.47 ha (67.70%)	40.05 ha (67.01%)	41.39 ha (69.26%)	40.20 ha (67.26%)	Initial: 59.77 ha
Remaining Primary Forest Ratio	32.30% (Feasible)	32.99% (Feasible)	30.74% (Feasible)	32.74% (Feasible)	Mandatory RFC >= 30.00%
Gross Timber Revenue (PEN)	2,067,155.66	2,065,103.95	1,998,573.64	2,064,844.60	Max: 3,099,712.93 PEN
Total Extraction Cost (PEN)	899,522.85	899,929.91	897,075.09	898,488.01	Max: 900,000.00 PEN
Budget Utilization (%)	99.95%	99.99%	99.68%	99.83%	Compliant (Phard = 0)
Economic Excess Penalties (PEN)	124,723.22	104,141.84	112,481.64	124,783.75	Soft ecological penalty
Net Economic Return (F(X) in PEN)	1,042,909.59	1,061,032.19	989,016.90	1,041,572.84	Objective function
Restricted Species Limit Violations	Palo Sangre (+41) Aguano Pashaco (+51)	Palo Sangre (+33) Aguano Pashaco (+49)	Palo Sangre (+32) Aguano Pashaco (+59)	Palo Sangre (+38) Aguano Pashaco (+57)	17 preserved, 2 penalized
Hard Ecological Constraints Status	Feasible (Phard = 0)	Feasible (Phard = 0)	Feasible (Phard = 0)	Feasible (Phard = 0)	100% legal compliance
Convergence Iteration/Budget	719/1000	290/1000	461/1000	312/1000	1000 iterations

## Data Availability

The data supporting the findings of this study, including parcel records, adjacency matrices, generation scripts, and algorithm implementations, are openly available in a GitHub repository at https://github.com/SebastianGrajeda28/Optimizing-tree-selection-using-bio-inspired-algorithms (accessed on 28 September 2026).

## Abbreviations

The following abbreviations are used in this manuscript:

ABC	Artificial Bee Colony
BFS	Breadth-First Search
GA	Genetic Algorithm
GRASP	Greedy Randomized Adaptive Search Procedure
TS	Tabu Search
SA	Simulated Annealing
